# Dapagliflozin regulates chondrocyte homeostasis and protects against osteoarthritis via targets AMPKα and SGLT2

**DOI:** 10.1038/s41420-026-03016-y

**Published:** 2026-03-19

**Authors:** Kaiwen Liu, Zedi Li, Cheng Wang, Houyi Sun, Jie Zhao, Meng Si

**Affiliations:** 1 Department of Orthopedic, Qilu Hospital of Shandong University, Jinan, China; 2 Department of Gastroenterology, Qilu Hospital of Shandong University, Jinan, China; 3 Department of Ophthalmology, Shandong Provincial Hospital Affiliated to Shandong First Medical University, Jinan, China

**Keywords:** Pharmacology, Diseases

## Abstract

Osteoarthritis (OA) is a degenerative joint disease characterized by progressive cartilage degradation and a complex pathogenesis. Degenerated chondrocytes exhibit an imbalance between catabolism and anabolism, leading to cartilage matrix loss. Currently, there are no effective clinical therapies to halt or reverse this degeneration. This study investigated the therapeutic potential of Dapagliflozin (DAPA) for OA. We demonstrated that DAPA exerts protective effects on cartilage explants from patients with OA as well as in surgically induced OA models in mice. In vitro studies revealed that DAPA ameliorates OA by restoring chondrocyte metabolic homeostasis. Transcriptome sequencing showed that DAPA activated the AMP-activated protein kinase (AMPK) signaling pathway while suppressing MAPK signaling. Mechanistically, AMPKα was identified as a novel target of DAPA. DAPA alleviated excessive catabolism by targeting both AMPKα and SGLT2, while promoting anabolic processes through AMPKα activation. Furthermore, DAPA rescued impaired autophagy caused by SGLT2 upregulation in degenerated chondrocytes. Our findings demonstrated that DAPA regulates cartilage metabolism by concurrently modulating AMPKα and SGLT2, underscoring the therapeutic promise of combined AMPK activation and SGLT2 inhibition in OA treatment.

**Figure:**
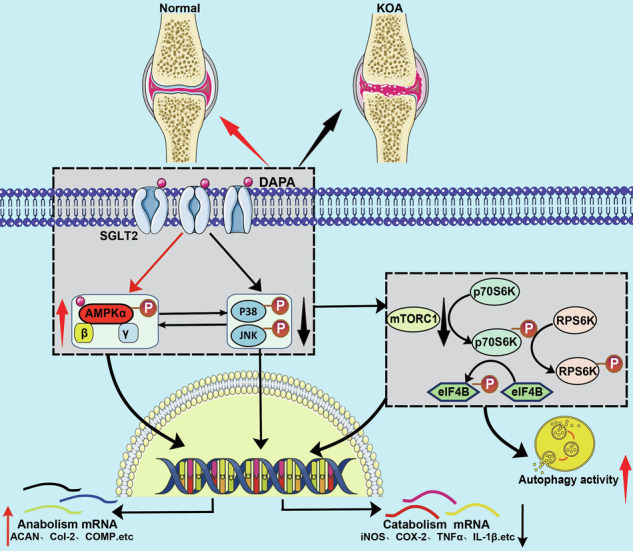
Mechanism of DAPA in treating osteoarthritis. Created with BioRender.com.

Mechanism of DAPA in treating osteoarthritis. Created with BioRender.com.

## Introduction

Osteoarthritis (OA) is a chronic degenerative joint disease that affects over 500 million individuals and is a major cause of disability worldwide [1, 2]. Cartilage degeneration plays a critical role in the pathogenesis of OA and is primarily characterized by cartilage tissue destruction and the loss of chondrocytes [3, 4]. Effective treatment options for early OA are still lacking, resulting in patients undergoing joint replacement surgery at the end-stage [5]. Currently, the pharmacological treatment of OA primarily focuses on pain relief, underscoring the urgent need to develop new therapies that target both symptom alleviation and joint structural modification [6].

Sodium-glucose cotransporter 2 (SGLT2) is a membrane protein widely distributed in various tissues and organs of the human body, including the kidneys, intestines, and heart [7, 8]. Its primary function is to promote glucose reabsorption by cotransporting sodium and glucose [9]. Increased SGLT2 expression under pathological conditions may lead to a series of adverse effects, including enhanced inflammatory responses, increased oxidative stress, and suppression of autophagy. SGLT2 inhibitors are a class of drugs widely used in clinical practice that have demonstrated significant clinical efficacy in the treatment of type 2 diabetes, diabetic kidney disease, and heart failure [10]. Mechanistically, SGLT2 inhibitors can inhibit the expression of pro-inflammatory cytokines in various pathological states, thereby exerting multiple biological effects, including reducing inflammation, alleviating cellular oxidative damage, promoting autophagy, and delaying cellular aging [11–13]. It is noteworthy that these biological effects play a significant role in the pathological process of degenerated osteoarthritic chondrocytes [14], suggesting that SGLT2 inhibitors represent a promising candidate strategy for therapeutic intervention in OA. Furthermore, previous studies have shown that various SGLT2 inhibitors activate the AMP-activated protein kinase (AMPK) signaling pathway [15, 16]. The AMPK signaling pathway is a major regulator of energy balance and metabolism [17]. AMPK activation is also believed to help maintain cartilage homeostasis and plays an important role in the treatment of OA [18, 19]. Although SGLT2 inhibitors have demonstrated potential to exert effects through pathways such as AMPK in various diseases, their direct role and specific targets in OA remain incompletely understood.

Dapagliflozin (DAPA) is a widely clinically deployed SGLT2 inhibitor. Interestingly, a recent studies have found that DAPA inhibits IL-1β-mediated endoplasmic reticulum stress-induced chondrocyte apoptosis by activating Sirt1 in rat chondrocytes [20]. However, in our study, DAPA did not bind directly to SIRT1. Therefore, the specific targeting mechanisms and protective effects of DAPA on OA remain unclear. Given the potential mechanisms of action of DAPA and the aforementioned studies, we consider DAPA to be a promising therapeutic drug for OA. Therefore, the aims of this study were to explore the protective effects of DAPA on OA and the underlying molecular mechanisms. We conducted a comprehensive analysis using cellular and animal models to confirm the potential clinical application of DAPA in the treatment of OA. Additionally, we identified AMPKα and SGLT2 as direct targets through which DAPA exerts its chondroprotective effects, demonstrating that both are essential for DAPA’s regulation of chondrocytes.

## Results

### DAPA alleviates IL-1β-mediated chondrocyte catabolism and enhances chondrocyte anabolism

**Fig. 1:**
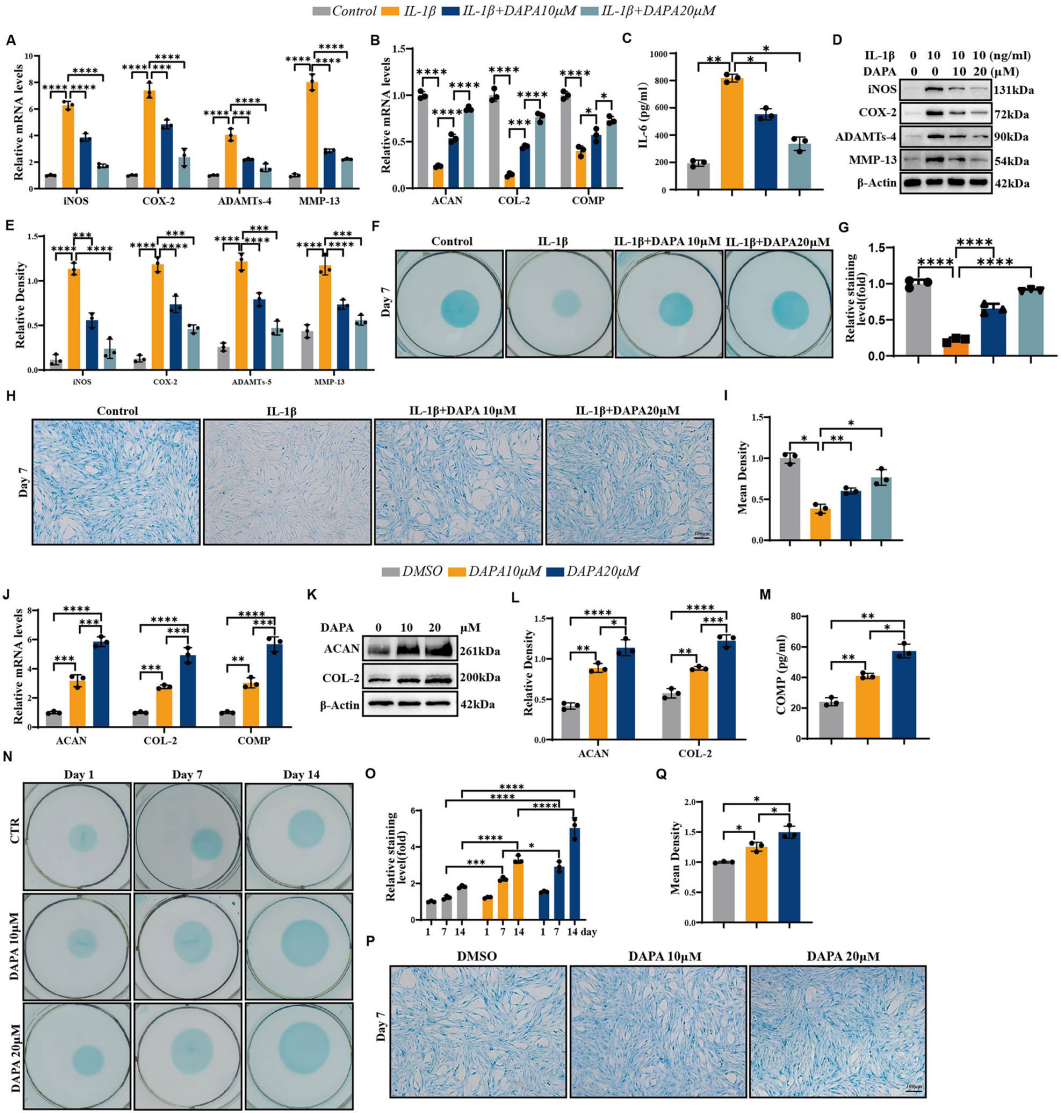
DAPA alleviates IL-1β-mediated chondrocyte catabolism and enhances chondrocyte anabolism. **A**, **B** Human normal chondrocytes were incubated at different concentrations of DAPA and stimulated with or without IL-1β (10 ng/mL) for 24 h. Real-time PCR was performed to examine the mRNA expression of iNOS, COX-2, ADAMTs-4, MMP-13, ACAN, COL2A1, and COMP (*n* = 3). **C** Expression of IL-6 in the cell supernatant was determined using ELISA (*n* = 3). **D** Human normal chondrocytes were treated with or without IL-1β and DAPA for 48 h. The protein expression of iNOS, COX-2, MMP-13, and ADAMTs-4 was determined using Western Blotting (*n* = 3). **E** Quantification of (**D**). **F–I** Human normal chondrocytes were treated at different concentrations of DAPA and stimulated with or without IL-1β, followed by Alcian Blue staining. Scale bar=100 µm. Quantification of Alcian Blue staining by Image J (*n* = 3). **J** Human normal chondrocytes were incubated at different concentrations of DAPA for 24 h. Real-time PCR was performed to examine the mRNA expression of ACAN, COL2A1, and COMP (*n* = 3). **K** Human normal chondrocytes were incubated at different concentrations of DAPA for 48 h. Western Blot was performed to examine the protein level of ACAN and COL2A1. **L** Quantification of (**K**) by Image J (*n* = 3). **M** Expression of COMP in the cell supernatant was determined using ELISA (*n* = 3). **N** Human normal chondrocytes were incubated in the absence or presence of 10 µM or 20 µM DAPA for 1, 7, 14 days, followed by Alcian blue staining. **O** Quantification of (**N**). (*n* = 3). **P** Human normal chondrocytes were treated at different concentrations of DAPA, followed by Alcian Blue staining. Scale bar=50 µm. **Q** Quantification of (**P**). (*n* = 3). Statistical analysis was performed using one-way ANOVA with Tukey’s post hoc test. Significant differences are indicated as follows: *P < 0:05, **P < 0:01, ***P < 0:001, and ****P < 0:0001.

DAPA can inhibit the expression of pro-inflammatory cytokines under various pathological conditions [13]. We aimed to determine whether DAPA suppressed catabolic metabolism in chondrocytes activated by pro-inflammatory cytokines. We selected IL-1β as the pro-inflammatory factor to investigate the effects of DAPA since it plays a key role in the pathogenesis of OA [21]. Human normal chondrocytes were treated with IL-1β and DAPA for 24 h. As shown in Fig. 1A, B, compared to the IL-1β group, DAPA treatment significantly downregulated the mRNA expression of catabolism markers ADAMTS4 and MMP13, as well as inflammatory factors iNOS and COX2, while upregulating the mRNA expression of anabolism markers ACAN, COL2A1 and COMP. Additionally, the ELISA (Fig. 1C). Western blotting (Fig. 1D, E) results also showed significant improvements in the protein levels of catabolic genes (ADAMTS4 and MMP13) and inflammatory factors (iNOS, COX2, and IL-6) in the DAPA treatment group. Immunofluorescence (Fig. S1A, B) and Alcian blue (Fig. 1F–I) staining of chondrocytes demonstrated that DAPA treatment reversed the inhibition of anabolism metabolism in chondrocytes caused by IL-1β. These results indicated that DAPA effectively inhibits the imbalance between anabolism and catabolism induced by IL-1β in chondrocytes.

We assessed whether DAPA induces chondrogenesis to understand the effects of DAPA on chondrocytes comprehensively. We first treated normal human chondrocytes with different concentrations of DAPA, and examined the mRNA expression of the anabolic markers ACAN, COL2A1, and COMP after 24 h. The results showed that DAPA promoted the transcription of anabolic genes in the chondrocytes in a concentration-dependent manner (Fig. 1J). Additionally, we assessed the protein levels of chondrogenic markers. ELISA, western blotting, and immunofluorescence analyses indicated that the protein expression of ACAN, COL2A1, and COMP was significantly upregulated after DAPA treatment (Fig. 1J–M**&** Fig. S1C, D). Alcian blue staining also confirmed the promoting effect of DAPA on chondrocyte anabolism (Fig. 1N–Q).

### DAPA regulates cartilaginous tissue and chondrocyte metabolism ex-vivo

**Fig. 2:**
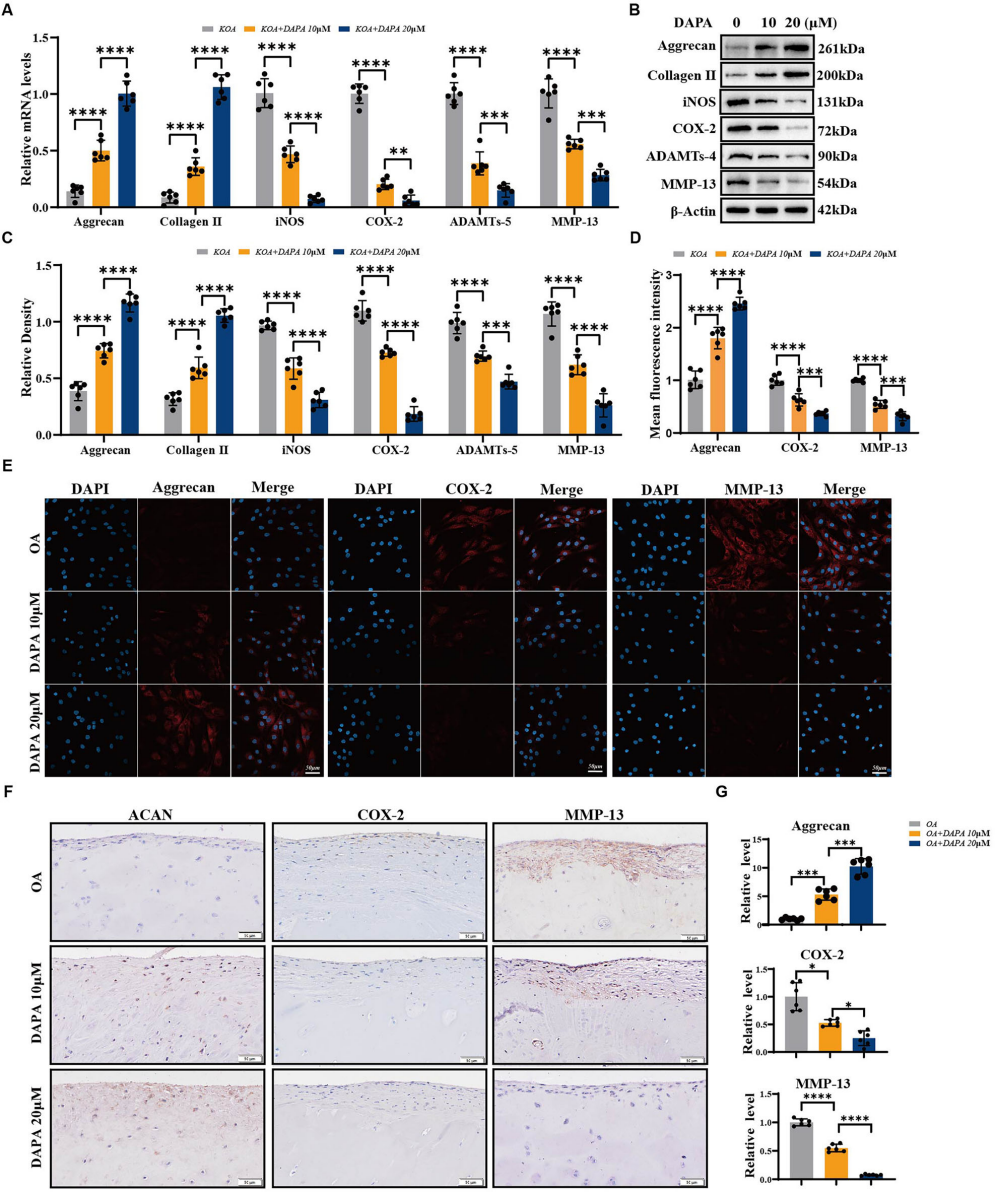
DAPA regulates cartilaginous tissue and chondrocyte metabolism ex-vivo. **A** Cartilage tissues from patients with OA were treated with DAPA (10, 20 µM) for 24 h followed by qRT-PCR analysis to detect the mRNA level of ACAN, COL2A1, iNOS, COX-2, ADAMTs-4, and MMP-13 (*n* = 6). **B** Cartilage tissues from patients with OA were treated with DAPA (10, 20 µM) for 48 h. Western blot was used to detect the protein level of ACAN, COL2A1, iNOS, COX-2, ADAMTs-4, and MMP-13. **C** Quantification of (**B**) by Image J (*n* = 6). **D**, **E** Chondrocytes were isolated from patients with OA, treated with DAPA (10, 20 µM) for 48 h, and analyzed by immunofluorescence staining of ACAN, COX-2, and MMP13. Scale bar=50 µm. Quantification of immunofluorescence staining by Image J (*n* = 6). **F** Immunohistochemistry of ACAN, COX-2, and MMP13 in cartilage tissues from patients with OA treated with DAPA (10, 20 µM) for 48 h. Scale bar=50 µm. **G** Quantification of Immunohistochemical staining in (**F**) by Image J (*n* = 6). Statistical analysis was performed using one-way ANOVA with Tukey’s post hoc test. Significant differences are indicated as follows: *P < 0.05, **P < 0.01, and ***P < 0.001.

Chondrocytes of patients with OA are in a state of imbalance between anabolism and catabolism [4]. We extracted chondrocytes from the knee joints of patients with OA and treated them with different DAPA concentrations to explore the therapeutic effects of the drug in OA. As shown in Fig. 2A, DAPA increased the mRNA expression levels of the anabolic markers ACAN and COL2A1 in a concentration-dependent manner compared to those in the control group. DAPA significantly reduced the mRNA expression of inflammatory factors (iNOS and COX2) and catabolic markers (MMP13 and ADAMTs4) in OA chondrocytes. Additionally, we extracted proteins from OA chondrocytes treated with DAPA for 48 h, and western blotting confirmed that DAPA upregulated the protein levels of ACAN and COL2 and downregulated the expression of inflammatory factors (iNOS and COX2) and catabolic markers (MMP13 and ADAMTs4) (Fig. 2B, C). Immunofluorescence experiments further confirmed that DAPA increased ACAN expression and decreased COX-2 and MMP13 (Fig. 2D, E). Cartilage tissue from knee replacement patients was treated with different concentrations of DAPA. Figure 2F, G shows that, at the histological level, DAPA treatment increased ACAN protein levels in OA cartilage tissue and significantly reduced the levels of COX-2 and MMP13. These results revealed that DAPA promoted anabolic metabolism and inhibited catabolic metabolism in the cartilage tissue of patients with OA in a concentration-dependent manner.

### DAPA protects against OA in surgically induced OA models

**Fig. 3:**
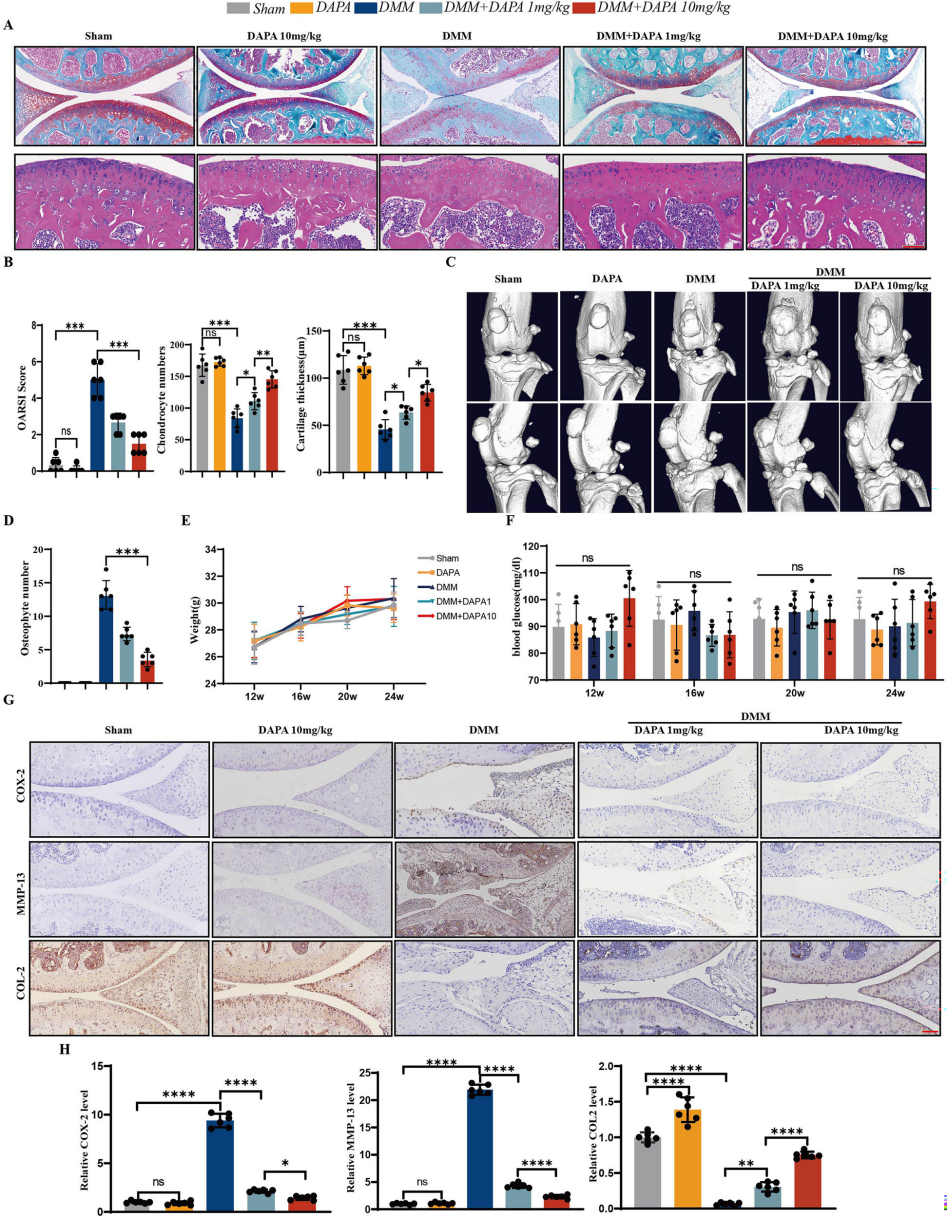
DAPA protects against OA in surgically induced OA models. **A** Representative images of safranin O/Fast green stained (Scale bar=200 µm) and H&E staining (Scale bar=100 µm) sections of knee joints from DMM surgery mouse models treated with DMSO or DAPA (10, 20 µM) for 12 weeks. **B** Quantitative analysis of OARSI scoring based on Safranin O/Fast Green staining, subchondral plate thickness, and chondrocyte number based on H&E staining (*n* = 6). **C**, **D** Representative three-dimensional micro-CT images of pathological structural changes in mouse knee joints 12 weeks after surgery. Quantification of osteophyte numbers (*n* = 6). **E**, **F** Body weight and fasting blood glucose levels were monitored in mice at baseline (post-surgery), and at 4, 8, and 12 weeks postoperatively (*n* = 6). **G** Immunohistochemical staining of COX-2, MMP13, and Collagen II in knee-joint sections from mice treated with DMSO or DAPA (10, 20 µM) for 12 weeks. Scale bar=100 µm. Significant differences are indicated as follows. **H** Quantification of Immunohistochemical staining in (**F**). Statistical analyses were performed as follows: the Kruskal-Wallis test with Dunn’s post-hoc comparisons was used for OARSI score and Figures D, while one-way analysis of variance (ANOVA) with Tukey’s post-hoc comparisons was applied for the remaining data. Significant differences are indicated as follows: *P < 0.05, **P < 0.01, and ***P < 0.001; ns: not significant.

We destabilized the medial meniscus (DMM) in 12-week-old male C57BL/6 J mice and initiated oral administration of DAPA or vehicle control 3 days post-surgery to evaluate the therapeutic effects of DAPA on osteoarthritis. Blood glucose levels and body weights were measured in all experimental groups immediately, as well as 4, 8, and 12 weeks after surgery. No statistically significant differences were observed between the groups (Fig. 3E-F).

Histological analysis of the knee joints was performed 12 weeks after the DMM surgery. As expected, severe cartilage degeneration with significantly elevated Osteoarthritis Research Society International (OARSI) scores was observed in the DMM group at 12 weeks postoperatively. DAPA treatment markedly attenuated the DMM-induced cartilage tissue loss (Fig. 3A, B). Hematoxylin and Eosin (H&E) staining of knee joint sections revealed reduced chondrocyte numbers in the DMM group compared to the sham group. In contrast, DAPA administration significantly increased chondrocyte density and cartilage layer thickness, demonstrating its inhibitory effect on cartilage degradation in the DMM-induced injury model (Fig. 3A, B).

Furthermore, H&E staining analysis of the subchondral bone thickness showed sclerotic changes characterized by thickened subchondral bone plates and trabeculae at 12 weeks post-DMM surgery. DAPA treatment significantly mitigated subchondral bone thickening, indicating its therapeutic potential against subchondral sclerosis (Fig. S2A). Abnormal subchondral bone remodeling, a hallmark of osteoarthritis, plays a critical role in providing nutritional and mechanical support to cartilage [22, 23]. Significant compositional and structural alterations occur in the subchondral bone during osteoarthritis progression. Given the pivotal role of osteoclasts in early-stage subchondral bone remodeling during OA, we investigated whether DAPA exerts subchondral protection through osteoclast suppression [24]. DAPA significantly downregulated the mRNA and protein expression of osteoclast markers, including NFATc1, c-Fos, MMP9, ACP5, and Cathepsin K (CTSK), in RANKL (100 ng/mL)-stimulated bone marrow-derived macrophages (BMDMs) (Fig. S2B–D). These results indicated that DAPA inhibited c-Fos expression, thereby suppressing RANKL-mediated NFATc1 activation. Immunofluorescence staining confirmed that DAPA treatment blocked NFATc1 nuclear translocation (Fig. S2E). Collectively, these findings demonstrated that DAPA suppressed osteoclast formation and function by downregulating NFATc1 and its downstream targets. Subsequent tartrate-resistant acid phosphatase (TRAP) staining of the mouse tibia and BMDMs confirmed the inhibitory effect of DAPA on osteoclastogenesis (Fig. S2F, G).

Micro-computed tomography (µCT) analysis revealed that DAPA significantly reduced osteophyte formation induced by DMM surgery at 12 weeks (Fig. 3C, D). Immunohistochemical staining showed that DMM surgery markedly decreased the expression of the anabolic marker COL2A1 in the cartilage, which was substantially reversed by DAPA treatment. DAPA effectively suppressed the upregulation of catabolic (MMP13) and inflammatory (COX2) markers in chondrocytes following DMM surgery (Fig. 3G, H). Overall, these results demonstrated that DAPA significantly attenuated DMM-induced osteoarthritis progression.

### DAPA Regulates chondrocyte function in an AMPK and MAPK pathway-dependent manner

**Fig. 4:**
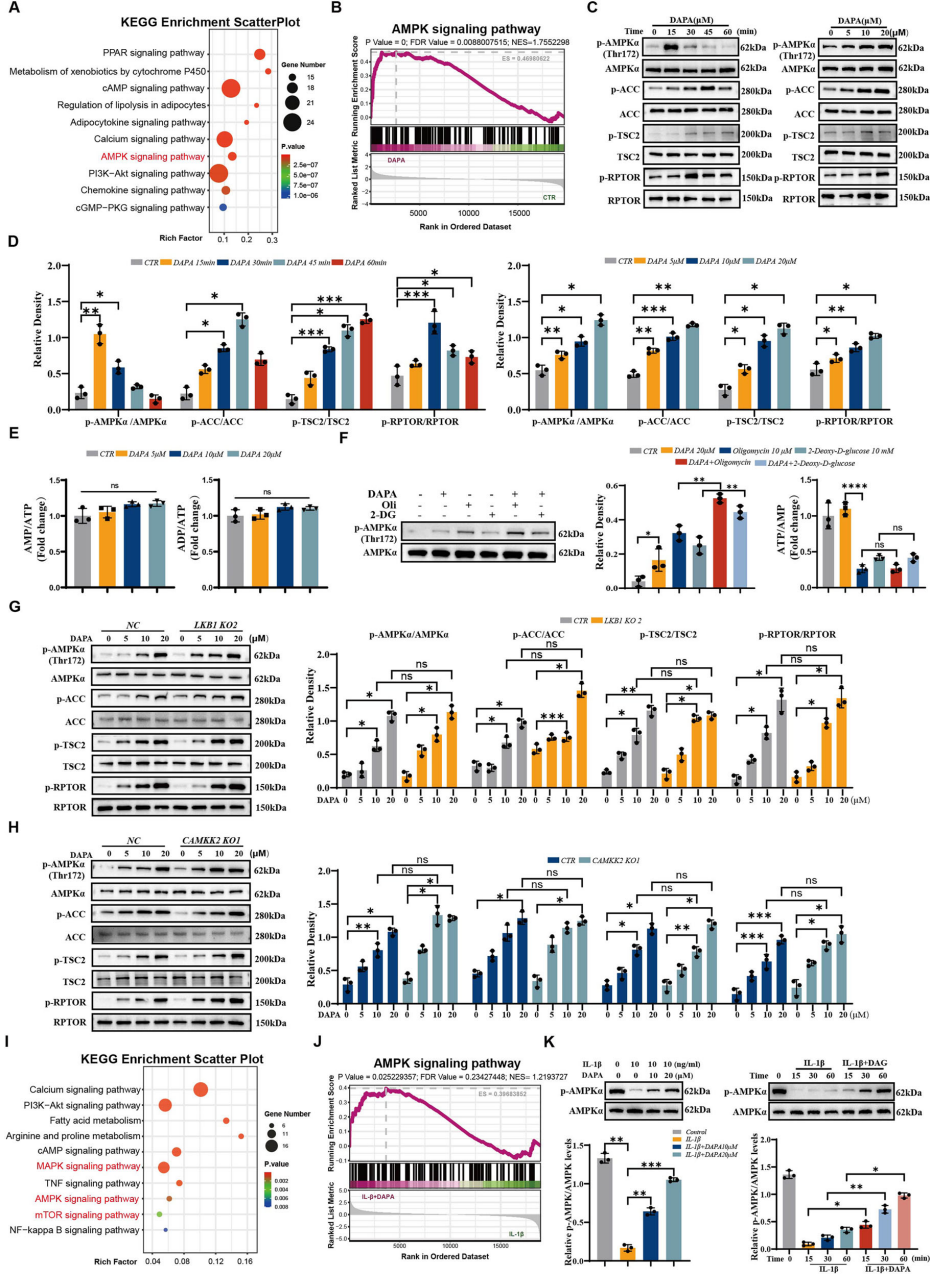
DAPA activates the AMPK signaling pathway. **A** KEGG analysis of differentially expressed mRNAs in rat chondrocyte. **B** Gene set enrichment analysis (GSEA) plot of differentially expressed genes in AMPK pathway between DMSO- and DAPA-treated rat chondrocyte. **C** Human normal chondrocytes were incubated with DAPA (20 μM) for different lengths of time or different concentrations of DAPA (0, 5, 10, 20 μM) for 30 min after which total proteins were extracted and the expressed of the AMPK, p-AMPK, ACC, p-ACC, TSC2, p-TSC2, RPTOR, and p-RPTOR was assessed by Western blotting. **D** Quantification of Western blotting results (*n* = 3). **E** AMP: ATP and ADP:ATP ratios were measured in human normal chondrocytes treated with DAPA at concentrations of 0, 5, 10, or 20 μM for 12 h. **F** Representative immunoblotting of p-AMPKα and AMPKα in the human normal chondrocytes from different groups and quantification of p-AMPKα/AMPKα. ATP:AMP ratios were measured in different groups. **G** Immunoblot analysis of AMPK, ACC, TSC2, and RPTOR signaling activation in control and LKB1-knockout C28 I2 cells treated with DAPA (0, 5, 10, 20 µM) for 30 min. **H** Immunoblot analysis of AMPK, ACC, TSC2, and RPTOR signaling activation in control and CAMKK2-knockout C28 I2 cells treated with DAPA (0, 5, 10, 20 µM) for 30 min. **I** KEGG analysis of changed genes in rat chondrocyte stimulated with IL-1β (10 ng/mL) and treated with or without DAPA (10 µM) for 24 h. **J** GSEA results show upregulation of the AMPK signaling pathway after treated with IL-1β and Dapagliflozin. **K** Human normal chondrocytes were treated with IL-1β (10 ng/mL) and/or Dapagliflozin for different concentrations or different time point, followed by immunoblot analysis of AMPK and p-AMPK. Quantification of Western blotting results (*n* = 3). Statistical significance was determined by one-way (Fig. D-F, K) or two-way (Fig. G, H) ANOVA, followed by Tukey’s post hoc test for multiple comparisons. Significant differences are indicated as follows: *P < 0.05, **P < 0.01, and ***P < 0.001; ns: not significant.

We performed RNA sequencing (RNA-seq) of rat chondrocytes treated with DMSO and DAPA to determine the mechanism by which DAPA promotes anabolism in chondrocytes. The results showed that DAPA up- and downregulated 502 and 441 genes, respectively (Fig. S3). Kyoto Encyclopedia of Genes and Genomes (KEGG) analysis of the RNA-seq data revealed that the AMPK signaling pathway was significantly enriched in chondrocytes treated with DAPA compared to those in the DMSO treatment group (Fig. 4A). Additionally, gene set enrichment analysis (GSEA) indicated that the AMPK signaling pathway was activated in chondrocytes following DAPA treatment (Fig. 4B), suggesting that DAPA may exert its effects through the AMPK signaling pathway. We treated normal human chondrocytes with varying concentrations and durations of DAPA, followed by western blot analysis of total and phosphorylated AMPK levels to investigate whether DAPA activates anabolism in chondrocytes via the AMPK signaling pathway. As shown in Fig. 4C, D, DAPA induced AMPK phosphorylation in a dose- and time-dependent manner. Concurrently, DAPA enhanced the phosphorylation of Acetyl-CoA Carboxylase (ACC), Tuberin (TSC2), and Regulatory-associated protein of mTOR (RPTOR) in a dose- and time-dependent manner, which was consistent with AMPK pathway activation.

AMPK is typically activated by increased AMP:ATP and ADP:ATP ratios; however, DAPA treatment did not alter these ratios (Fig. 4E). We used an energy stress model to detect changes in the ATP:AMP ratio while monitoring AMPK phosphorylation levels, thereby distinguishing between the direct and indirect activation mechanisms of DAPA and further validating this hypothesis. The results showed that oligomycin and 2-DG, as positive controls for energy stress, activated AMPK phosphorylation by reducing the ATP:AMP ratio. In contrast, DAPA treatment enhanced AMPK phosphorylation without changing the ATP:AMP ratio, indicating that this increase in phosphorylation was not driven by energy stress. In both the oligomycin + DAPA and 2-DG + DAPA treatment groups, AMPK phosphorylation was further enhanced, yet the ATP:AMP ratio showed no statistically significant difference compared to the oligomycin- or 2-DG-alone groups. This confirmed that the activation effect of DAPA operates independently of the energy-sensing pathway (Fig. 4F). LKB1 and CAMKK2 are primarily responsible for AMPK phosphorylation and activation. However, knockout of LKB1 or CAMKK2 did not alter DAPA-induced AMPK activation, as demonstrated by the increased phosphorylation of AMPK, ACC, TSC2, and RPTOR (Fig. S4A, B and Fig. 4G, H). Collectively, these data demonstrated that DAPA directly enhances AMPK activity rather than stimulating upstream kinases or altering AMP:ATP and ADP:ATP ratios.

The AMPK signaling pathway plays a crucial role in regulating homeostasis of anabolism and catabolism in chondrocytes [25, 26]. Notably, the DAPA-mediated expression of anabolic marker genes (e.g., COL2A1, ACAN, and COMP) was completely suppressed by the AMPK inhibitor Compound C (Fig. S5A–C), confirming that DAPA enhanced chondrocyte anabolic function by activating the AMPK pathway. Furthermore, the anti-catabolic effect of DAPA on chondrocytes was partially retained after treatment with Compound C (Figure S5D–F), indicating that this effect partially relies on AMPK pathway activation. However, it may also synergistically enhance its overall effect against inflammation-induced catabolism by triggering other AMPK-independent anti-inflammatory mechanisms.

We performed RNA-seq on rat chondrocytes treated with IL-1β and DAPA to determine the anti-inflammatory mechanism of DAPA in cartilage degeneration. The volcano plot showed that 133 genes were upregulated and 211 genes were downregulated compared to the IL-1β treatment group (Fig. S6A). The RNA-seq results showed that compared to the IL-1β treatment group, the MAPK and AMPK signaling pathways were significantly enriched in chondrocytes treated with DAPA (Fig. 4I). GSEA further indicated that the MAPK signaling pathway was suppressed, whereas the AMPK signaling pathway was activated after DAPA treatment (Fig. 4J and Fig. S6B). This suggested that DAPA exerts its effects through both the signaling pathways. We treated normal human chondrocytes with varying concentrations and durations of IL-1β and DAPA to investigate whether DAPA antagonizes IL-1β-mediated inflammatory responses via the MAPK and AMPK signaling pathways. As expected, DAPA significantly negated the phosphorylation of p38 and JNK1/2 induced by IL-1β while restoring AMPK phosphorylation inhibition caused by IL-1β (Fig. 4K and Fig. S6C, D). These results indicated that DAPA suppresses catabolism in chondrocytes by regulating the MAPK and AMPK signaling pathways.

### DAPA’s activation of AMPK is independent of SGLT2

Multiple biological activities of DAPA are mediated through its canonical target SGLT2. We examined whether DAPA-mediated AMPK activation requires SGLT2 expression to investigate the mechanism by which DAPA regulates the AMPK signaling pathway. To this end, we used CRISPR/Cas9 technology to establish an SGLT2 knockout (KO) chondrocyte monoclonal cell line (Fig. S7A). DAPA promoted the phosphorylation of AMPK, ACC, TSC2, and RPTOR in SGLT2-deficient C28I2 cells, indicating that SGLT2 inhibition did not compromise DAPA-induced AMPK activation (Fig. S7B–D).

The known target of DAPA, SGLT2, is associated with the inflammatory response and autophagy [27]. We aimed to determine whether the anti-catabolic and anabolic functions of DAPA depend on its known target SGLT2. Therefore, we used specific small interfering RNA(siRNAs) to knock down the expression of SGLT2 in human normal chondrocytes (Fig. S7E). We found that the inhibition of SGLT2 expression did not affect DAPA’s promotion of chondrocyte anabolism (Fig. S7F); however, it partially influenced DAPA’s suppression of IL-1β-induced cytokine release (Fig. S7G–I). In summary, these results indicated that the anabolic effect of DAPA is independent of SGLT2, whereas its anti-inflammatory effect is partially dependent on SGLT2.

### AMPK is a novel target of DAPA, and DAPA enhances AMPK activity by targeting the kinase domain of the AMPKα subunit

**Fig. 5:**
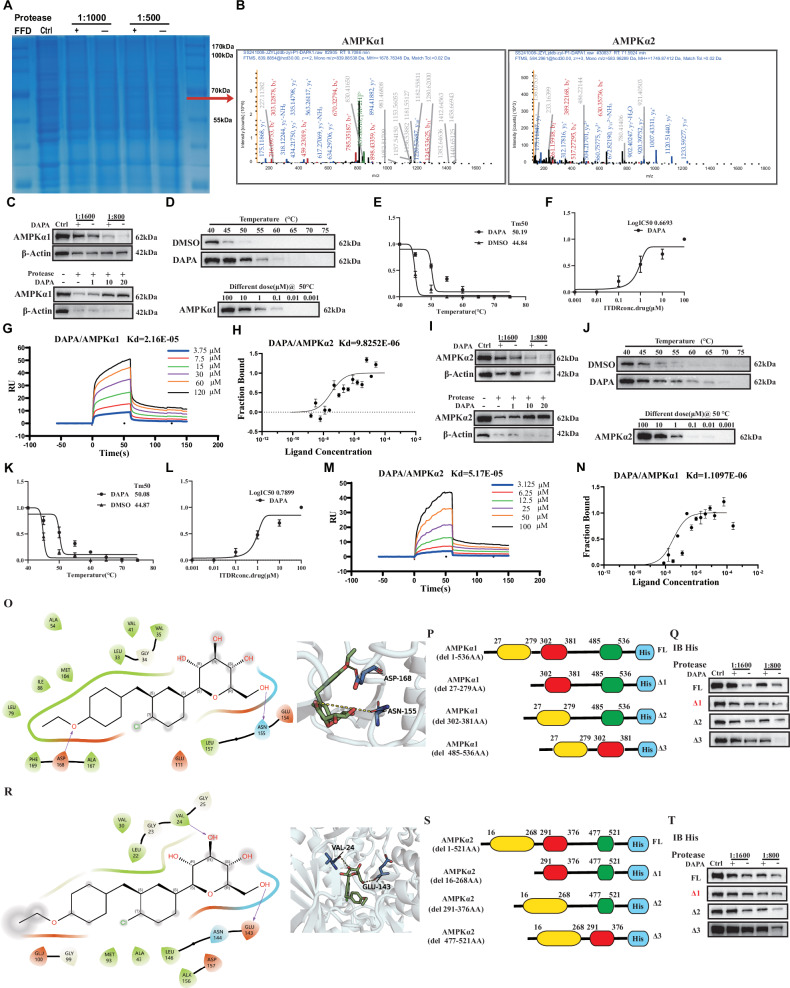
AMPKα is a novel target of DAPA. **A** Coomassie blue staining of SDS-PAGE gel of DARTS assay. The band with molecular weight between 60 kDa was protected by DAPA. **B** Mass spectrum of AMPKα1 and AMPKα2. **C** Human chondrocytes were digested with different concentrations of proteases with or without DAPA or digested with proteases with different concentrations of Dapagliflozin, after which AMPKα1 protein levels were determined using Western blotting. **D** CTESA assay with DAPA-treated chondrocyte, after which AMPKα1 protein levels were assessed by Western blotting. **E**, **F** Quantification of (**D**). **N** SPR assay for the affinity between DAPA and AMPKα1. **H** DAPA’s bond with AMPKα1 analyzed by MST assay. **I** Human chondrocytes were digested with different concentrations of proteases with or without DAPA or digested with proteases with different concentrations of DAPA, after which AMPKα2 protein levels were determined using Western blotting. **J** CTESA assay with DAPA-treated chondrocyte, after which AMPKα2 protein levels were assessed by Western blotting. **K, L** Quantification of (**J**). **M** SPR assay for the affinity between Dapagliflozin and AMPKα2. **N** DAPA’s bond with AMPKα2 analyzed by MST assay. **O** The visualized image of molecular docking between AMPKα1 and DAPA. **P** Schematic representation of expression constructs encoding full-length (FL) His-tagged AMPKα1 and its domain-deletion mutants (Δ1–Δ3). **Q** DARTS assay with serial deletion constructs encoding His-tagged mutants of AMPKα1. C28I2 cells were transfected with His-tagged mutants of AMPKα1 plasmids, as indicated. DARTS assay samples were detected by His antibody. **R** The visualized image of molecular docking between AMPKα2 and DAPA. **S** Schematic representation of expression constructs encoding full-length (FL) His-tagged AMPKα2 and its domain-deletion mutants (Δ1–Δ3). **(T**) DARTS assay for serial point mutants of AMPKα2. C28I2 cells were transfected with the plasmid expressing various His-tagged AMPKα2 mutants, as indicated. DARTS assay samples were detected by His antibody.

We conducted drug affinity responsive target stability (DARTS) assays to identify the direct targets of DAPA in activating the AMPK signaling pathway and treating cartilage degeneration [28, 29]. After separating the samples using SDS-PAGE, Coomassie blue staining revealed a prominent band in the molecular weight range of 55–75 kDa that was protected by DAPA (Fig. 5A). Mass spectrometry analysis of this band confirmed AMPKα1 and AMPKα2 as potential binding targets of DAPA (Fig. 5B).

We conducted western blot analysis on the samples from the DARTS experiment to further confirm whether AMPKα1 and AMPKα2 are indeed DAPA binding targets. The results indicated that in the DAPA-treated groups, the AMPKα1 and AMPKα2 proteins were protected from proteolytic digestion (Fig. 5C, I). Additionally, cellular thermal shift assay (CETSA) analysis revealed that DAPA prevented the denaturation of AMPKα1 and AMPKα2 at various temperatures compared to the control group, particularly at 50 °C. The melt curves demonstrated robust changes in the melting temperature (Tm) in the presence of DAPA, with Tm values of 50.19 °C for AMPKα1 and 50.08 °C for AMPKα2. We also conducted CETSA at 50 °C with different concentrations of DAPA, confirming that DAPA dose-dependently prevented the denaturation of AMPKα1 and AMPKα2, with EC50 values of 0.6693 and 0.7899, respectively. This experiment indicated that DAPA enhances the thermal stability of AMPKα1 and AMPKα2 within a specific temperature range, confirming the specific interaction between DAPA and AMPKα (Fig. 4D–F and Fig. 4J–L). Moreover, surface plasmon resonance and microscale thermophoresis technology demonstrated a robust affinity between DAPA and AMPKα1 and AMPKα2 (Fig. 4G, H and Fig. 4M, N). These results indicated a direct interaction between DAPA and the α subunits of AMPK, suggesting that AMPKα is a novel binding target for DAPA.

We conducted molecular docking simulations to understand how DAPA binds to AMPKα1 and AMPKα2. The results showed that DAPA forms a 3.0 Å hydrogen bond with AMPKα1 at ASN155 and a 5.1 Å hydrogen bond at ASP168. It also engaged in hydrophobic interactions with amino acids such as VAL35, LEU33, MET104, ILE88, VAL41, ALA54, LEU79, PHE169, ALA167, and LEU157 (indicated as green amino acids in the 2D diagram) (Fig. 5O). DAPA forms a 2.7 Å hydrogen bond with AMPKα2 at GLU143 and a 3.0 Å hydrogen bond at VAL24. It also interacted hydrophobically with amino acids, including VAL24, LEU22, VAL30, MET93, ALA43, LEU146, and ALA156 (also shown as green amino acids in the 2D diagram) (Fig. 5R). We designed several domain-deletion mutants of AMPKα1 and AMPKα2 to further delineate the binding site(s) of DAPA on AMPKα. Both AMPKα1 and AMPKα2 consist of three functional domains critical for their activity. Consequently, we constructed and validated three N-or C-terminal deletion mutants (designated Δ1, Δ2, and Δ3; Fig. 5P and Fig. 5S) targeting these domains. Subsequently, the binding of DAPA to these mutants was assessed using a DARTS assay, followed by western blot analysis. As shown in Fig. 5Q and Fig. 5T, DAPA provided protection similar to that observed with full-length AMPKα, specifically when bound to the kinase domain constructs [AMPKα1(27-279) and AMPKα2(16-268)]. This result identified the kinase domain of the AMPKα subunit as the binding domain for DAPA. Furthermore, molecular docking simulations also identified potential binding sites located within the kinase domain of AMPKα. Collectively, these experiments demonstrated that the kinase domain of AMPKα serves as the primary binding domain for DAPA.

We performed an in vitro AMPK kinase assay to confirm that DAPA activates the AMPK signaling pathway by directly binding to AMPK. As depicted in Fig. S8A, DAPA directly stimulated the kinase activity of recombinant AMPK in this cell-free system, leading to increased autophosphorylation of AMPKα. We further validated the binding of DAPA to other AMPK subunits using DARTS analysis. The results showed that DAPA does not bind to the β and γ subunits of AMPK (Fig. S8B–F). These results provided direct biochemical evidence that DAPA activates the AMPK pathway by directly engaging the AMPKα subunit.

Finally, although some studies have reported that DAPA exerts antiapoptotic effects on chondrocytes via SIRT1 activation, our DARTS and CETSA data demonstrated no direct binding between DAPA and SIRT1. This indicated that SIRT1 was not a direct target of DAPA (Fig. S8G).

### DAPA exerts chondroprotective effects through AMPKα and SGLT2

**Fig. 6:**
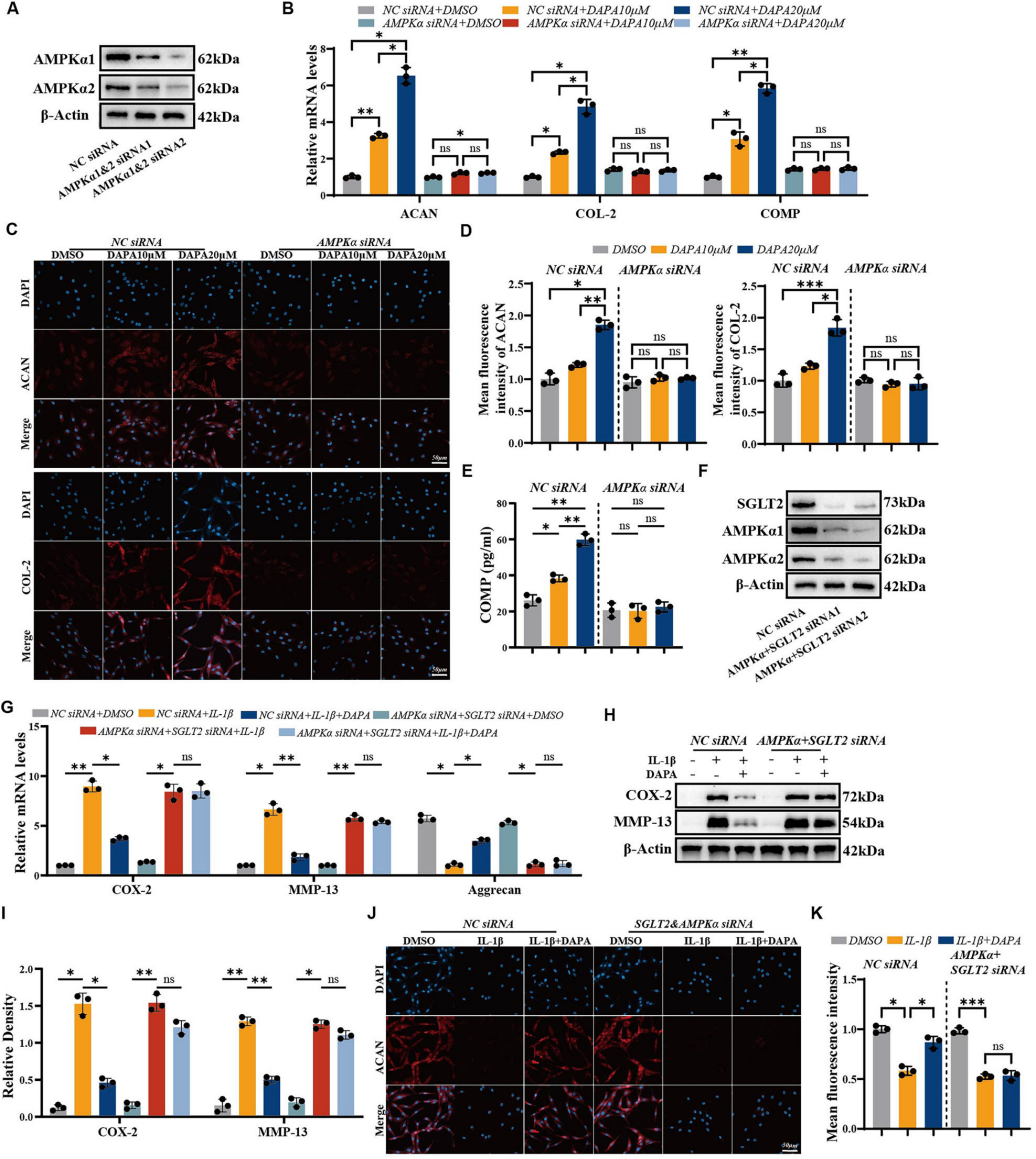
DAPA exerts chondroprotective effects through AMPKα and SGLT2. **A** Immunoblot analysis of AMPKα1 and AMPKα2 protein levels following siRNA-mediated knockdown. **B** RT-qPCR analysis of ACAN, COL2A1, and COMP mRNA expression in human normal chondrocytes treated with DAPA after AMPKα1/α2 knockdown (*n* = 3). **C** Immunofluorescence staining analysis of ACAN and COL2A1 expression in chondrocytes treated with DAPA after AMPKα1/α2 knockdown. Scale bar=50 μm. **D** Quantification of fluorescence intensity in (**C**). **E** ELISA quantification of COMP protein levels in cell supernatants (*n* = 3). **F** Immunoblot validation of AMPKα1, AMPKα2, and SGLT2 knockdown efficiency. **G** RT-qPCR analysis of COX-2, MMP-13, and ACAN mRNA in IL-1β-stimulated chondrocytes treated with/without DAPA following AMPKα1/α2/SGLT2 knockdown (*n* = 3). **H** Immunoblot analysis of COX-2 and MMP-13 protein levels in IL-1β-stimulated human normal chondrocytes treated with/without DAPA following AMPKα1/α2/SGLT2 knockdown (*n* = 3). **I** Densitometric quantification of immunoblot results in (**H**). **J** Immunofluorescence staining of ACAN in IL-1β-stimulated human normal chondrocytes treated with/without DAPA following AMPKα1/α2/SGLT2 knockdown (*n* = 3). Scale bar: 50 μm. **K** Quantification of ACAN fluorescence intensity in (**J**). Two-way ANOVA with Tukey’s multiple comparisons post-hoc test were used for statistical analysis. Statistical notation: *P < 0.05, **P < 0.01, ***P < 0.001; ns not significant.

Having identified AMPKα as the direct target through which DAPA modulates the AMPK signaling pathway, we next investigated whether DAPA’s anti-catabolic and pro-anabolic functions depend on AMPKα. siRNAs effectively knocked down AMPKα1 and AMPKα2 expression in human primary chondrocytes (Fig. 6A). Similar to results obtained with the AMPK inhibitor compound C, AMPKα knockdown negated DAPA-induced pro-anabolic effects. Consequently, treatment failed to upregulate the expression of anabolic marker genes, including ACAN, COL2, and COMP, in these cells (Fig. 6B). Immunofluorescence and ELISA data further confirmed that the protein levels of ACAN, COL2, and COMP remained unchanged (Fig. 6C–E), demonstrating that DAPA’s pro-anabolic activity is AMPKα-dependent.

Furthermore, DAPA-mediated suppression of IL-1β effects was partially reversed following AMPKα knockdown, leading to significant upregulation of catabolic (MMP13) and inflammatory (COX2) genes (Fig. S9A, B). Specifically, DAPA treatment only partially reduced IL-1β-induced COX-2 and MMP-13 expression and partially reversed IL-1β-mediated suppression of ACAN (Fig. S9C).

Subsequently, we simultaneously knocked down AMPKα1, AMPKα2, and SGLT2 in chondrocytes (Fig. 6F). DAPA’s inhibition of IL-1β activity was completely or near-completely canceled in the cells lacking both AMPKα and SGLT2 (Fig. 6G–K). These findings indicated that DAPA’s anti-inflammatory and anti-catabolic actions require a dual mechanism involving both AMPKα and SGLT2.

### DAPA targets SGLT2 to modulate MAPK/AMPK crosstalk, suppresses mTORC1 activity, and activates autophagy for chondroprotection

#### SGLT2 is associated with cartilage degeneration

**Fig. 7:**
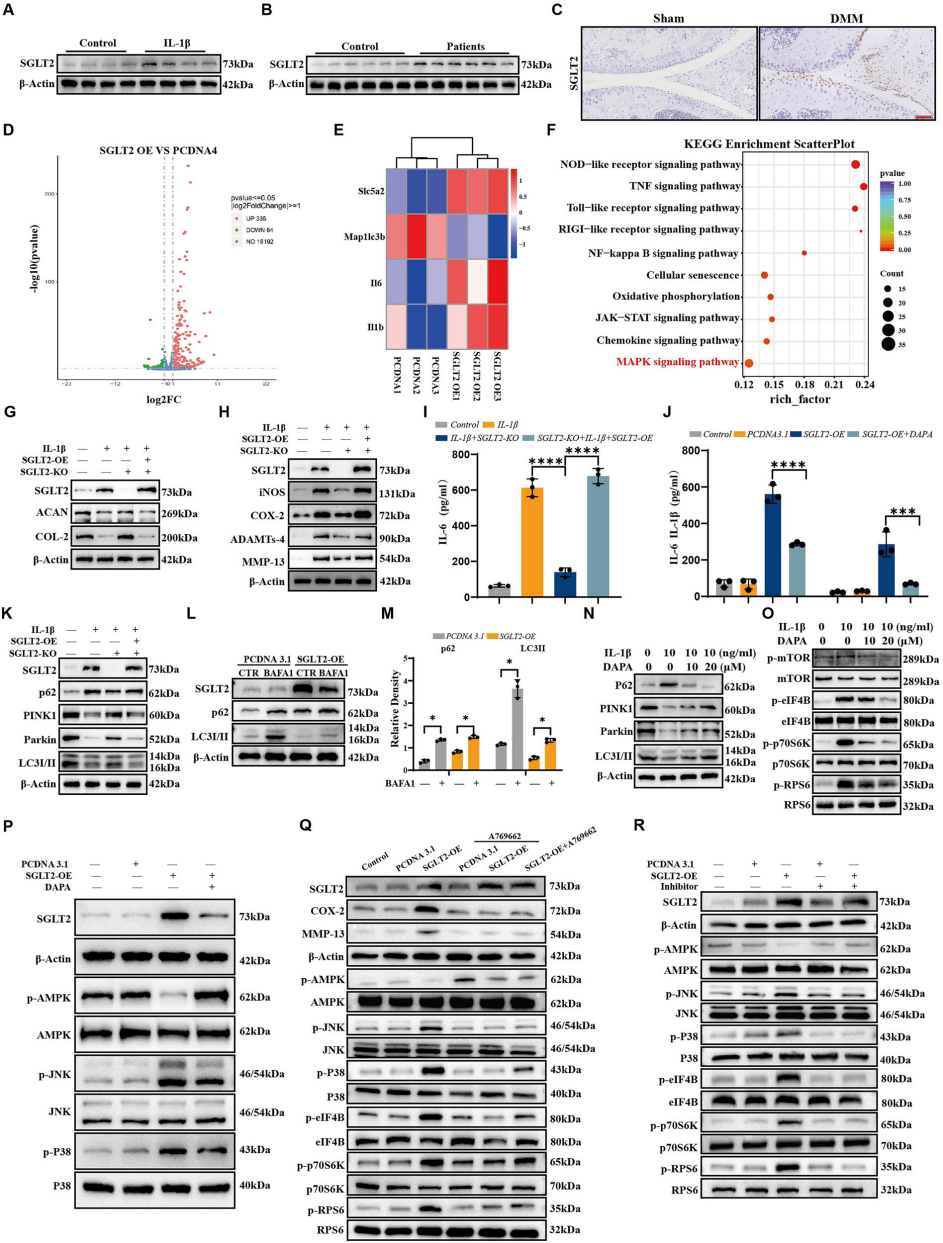
Dapagliflozin targets SGLT2 to modulate MAPK/AMPK crosstalk, suppresses mTORC1 activity, and activates autophagy for chondroprotection. **A** Western blot analysis of SGLT2 protein expression in human chondrocytes, with or without IL-1β treatment. Quantitative analysis of band intensity (*n* = 4). **B** Western blot detection of SGLT2 levels in chondrocytes from patients with chondral fractures and OA. Quantitative analysis (*n* = 6). **C** Immunohistochemical staining to detect SGLT2 levels in sham-operated and DMM model mice. Scale bars = 100 µm. Quantitative analysis of staining intensity (*n* = 6). **D** Volcano plot of mRNA sequencing. **E** Heatmap showing gene expression profiles comparing SGLT2-overexpressing (OE) and empty vector (pcDNA3.1) control groups. **F** KEGG pathway enrichment analysis of differentially expressed genes between experimental groups. **G**, **H** Western blot analysis of ACAN, COL2A1, iNOS, COX-2, ADAMTS-4, and MMP-13 expression in C28I2 cell. **I** The levels of IL-6 in C28I2 cell supernatant were detected by ELISA. **J** The levels of IL-1β and IL-6 in human chondrocytes supernatant were detected by ELISA. **K** Western blot analysis of p62, PINK1, Parkin, and LC3II expression in C28I2 cell. **L**, **M** Human chondrocytes were transfected with SGLT2 or control vectors, then treated with or without BAFA1. Protein levels of LC3-II and p62 were quantified by Western blotting. Quantitative analysis (*n* = 3). **N** Human chondrocytes treated with IL-1β (10 ng/mL) and/or DAPA for 48 h. Western blot analysis of p62, PINK1, Parkin, and LC3II expression. **O** Human chondrocytes treated with IL-1β (10 ng/mL) and/or dapagliflozin for 1 h. Western blot analysis of phosphorylation levels of mTOR, eIF4B, p70S6K, and RPS6. **P** Western blot analysis of SGLT2 and the phosphorylation of AMPK, JNK, and p38 in human chondrocytes. **Q** Human chondrocytes were pretreated with or without A769662, transfected with pcDNA3.1 or SGLT2, or transfected with SGLT2 followed by A769662 treatment. Protein expression of the inflammatory factor COX-2 and catabolic marker MMP-13, activation status of AMPK, JNK, and p38 signaling pathways, and mTORC1 activity were assessed by Western blotting. **R** Human chondrocytes were pretreated with or without BIRB796 and JNK-IN-8, transfected with pcDNA3.1 or SGLT2. Protein expression of the inflammatory factor COX-2 and catabolic marker MMP-13, activation status of AMPK, JNK, and p38 signaling pathways, and mTORC1 activity were assessed by Western blotting. Statistical analyses were performed using one-way ANOVA for Figures (**I**) and (**J**), and two-way ANOVA for Figure M, with Tukey’s post-hoc test applied for multiple comparisons. Statistical notation: *P < 0.05, **P < 0.01, ***P < 0.001, and ****P < 0.0001.

We examined the expression of SGLT2 in the cartilage of patients with and without OA to elucidate the role of SGLT2 in the progression of OA. Western blotting results showed that compared to normal non-degenerate cartilage tissue, SGLT2 protein levels were significantly elevated in the cartilage of patients with OA (Fig. 7A and Fig. S10A). Additionally, we observed a marked increase in SGLT2 expression in an in vitro model after stimulating normal chondrocytes with IL-1β for 48 h (Fig. 7B and Fig. S10B). Immunohistochemical analysis revealed a similar trend of increased SGLT2 protein levels in the cartilage of mice in the DMM surgery-induced OA model (Fig. 7C and Fig. S10C), suggesting that abnormal SGLT2 expression may be associated with the pathological progression of OA.

#### Transcriptome sequencing of SGLT2 overexpression

We transfected rat chondrocytes with SGLT2 overexpression plasmids and performed whole-transcriptome analyses to investigate the potential role of SGLT2 in chondrocyte damage. Differential gene expression screening revealed that SGLT2 overexpression led to the down- and upregulation of 64 and 335 genes, respectively (Fig. 7D). Among these, the mRNA levels of pro-inflammatory cytokines IL-6 and IL-1β were significantly elevated, while the expression of the autophagy marker MAP1LC3B was markedly suppressed (Fig. 7E). KEGG enrichment analysis (Fig. 7F) suggested that SGLT2 overexpression may regulate chondrocyte biological functions through multiple signaling pathways.

#### SGLT2 overexpression drives metabolic imbalance in chondrocytes

SGLT2 KO C28/I2 cells showed significantly upregulated expression of the anabolic markers ACAN and COL2A1 under IL-1β stimulation (Fig. 7G and Fig. S10D), while inhibiting the production of inflammatory factors (iNOS, COX2, and IL-6) and catabolic enzymes (MMP13 and ADAMTS4) (Fig. 7H, I and Fig. S10E). Notably, the transfection of KO chondrocytes with SGLT2 overexpression plasmids reversed these protective effects in a specific manner (Fig. 7G–I), further confirming that the loss of SGLT2 function can effectively counteract the metabolic imbalance in chondrocytes induced by inflammation. DAPA also significantly downregulated the release of inflammatory factors IL-6 and IL-1β promoted by SGLT2 overexpression in human chondrocytes (Fig. 7J).

#### SGLT2 overexpression suppresses autophagy, while DAPA restores autophagy via inhibition of mTORC1 activity

Given the central protective role of autophagy in cartilage homeostasis [30], we explored the regulatory mechanisms of SGLT2 in the autophagy pathway. As shown in Fig. 7K and Fig. S10F, IL-1β treatment significantly decreased the LC3B-II/LC3B-I ratio in SGLT2 KO C28/I2 cells, while increasing the accumulation of the autophagy substrate p62 and decreasing the expression of mitochondrial autophagy markers PINK1/Parkin. SGLT2 KO effectively restored autophagic flux, as evidenced by elevated LC3B-II levels, as well as accelerated p62 degradation and upregulation of PINK1/Parkin expression. The conversion of LC3-I to LC3-II decreased after transfection with the SGLT2 overexpression plasmid, PINK1 and Parkin expression decreased, and p62 expression increased.

We conducted a Bafilomycin A1 (BafA1) blocking experiment to clarify the dynamic changes in autophagic flux. BafA1 inhibits lysosomal acidification, preventing the fusion of autophagosomes with lysosomes and the degradation of LC3-II. As shown in Fig. 7L, M, in human chondrocytes, SGLT2-overexpressing cells displayed decreased LC3-II levels and increased p62 under basal conditions. The accumulation of LC3-II in these cells was significantly lower than that in the control group after treatment with BafA1, and the degree of p62 degradation blockage was reduced, indicating that SGLT2 overexpression impaired autophagic flux by inhibiting autophagosome biosynthesis.

Notably, DAPA intervention enhanced autophagy in a dose-dependent manner, and its effects were highly consistent with the SGLT2 KO phenotype (Fig. 7N and Fig. S10G). We explored changes in the mTOR signaling pathway using transcriptome analysis to further investigate the potential mechanism of DAPA-induced autophagy (Fig. 4D). Western blotting results showed that IL-1β stimulation significantly increased the phosphorylation levels of the mTORC1 downstream effectors S6, p70 S6K, and eIF4B (Fig. 7O and Fig. S10H). However, DAPA treatment inhibited these phosphorylation events in a dose-dependent manner, suggesting that DAPA relieves autophagy suppression by blocking the activation of the mTORC1 pathway.

### DAPA reverses SGLT2 overexpression-induced AMPK suppression and MAPK activation

We transfected human chondrocytes with an SGLT2 overexpression plasmid and experimentally validated the KEGG pathways To investigate the mechanism by which SGLT2 overexpression affects signaling pathways. Western blotting results revealed that SGLT2 overexpression significantly inhibited AMPK pathway activity while promoting the phosphorylation of key MAPK pathway molecules, JNK, and p38. This indicates that the AMPK and MAPK signaling pathways serve as key downstream regulatory nodes for SGLT2. However, DAPA treatment markedly reversed these effects (Fig. 7P).

### AMPK-MAPK crosstalk modulates mTORC1 activity

We treated human chondrocytes with SGLT2 overexpression (OE) using the AMPK agonist, A769662, to elucidate the mechanism by which AMPK signaling pathway activation counteracts SGLT2-mediated damage. The results showed that compared with the SGLT2-OE group, both the A769662 pre-treatment group (with AMPK pre-activation before SGLT2 overexpression) and the A769662 treatment group after SGLT2 overexpression significantly suppressed the abnormal elevation of inflammatory factors and catabolic markers induced by SGLT2-OE, reduced the phosphorylation levels of p38 and JNK1/2, and inhibited mTORC1 activity. This confirmed that AMPK phosphorylation counteracted SGLT2 overexpression-mediated MAPK signaling activation and enhanced mTORC1 activity (Fig. 7Q). Importantly, we found that the pharmacological inhibition of p38/JNK signaling reversed the SGLT2 overexpression-induced suppression of AMPK phosphorylation and activation of mTORC1 (Fig. 7R).

In summary, AMPK activation suppressed SGLT2 overexpression-induced MAPK signaling and mTORC1 activity. Furthermore, MAPK inhibition attenuated SGLT2 overexpression-mediated AMPK suppression. Critically, DAPA exerted chondroprotective effects by promoting chondrocyte autophagy through the targeted activation of AMPK and inhibition of SGLT2-mediated mTORC1 activation.

## Discussion

Osteoarthritis exhibits significant heterogeneity in its clinical presentation and pathological characteristics [27]. The interplay among multiple pathogenic factors ultimately leads to an imbalance between cartilage repair and degradation, thereby accelerating OA progression. A hallmark pathological feature of OA is the loss of cartilage, which is primarily characterized by the increased expression of matrix-degrading enzymes, inhibition of matrix molecule synthesis, and disruption of ECM remodeling [31]. DAPA is the first-line clinical drug used for the treatment of type 2 diabetes [32]. Increasing evidence has demonstrated that it has glucose-lowering properties and exerts anti-inflammatory, antioxidant, anti-aging, autophagy-enhancing, and anti-tumor effects in various cell types [33–37]. Recent studies have garnered widespread attention regarding the role of DAPA in the treatment [20]. Although one study found that DAPA can prevent OA progression and suggested that this effect might be related to the activation of Sirt1, which inhibits endoplasmic reticulum stress-mediated chondrocyte apoptosis in rats, our research confirmed that Sirt1 is not a direct target of DAPA and that there is no direct binding between the two. This suggested that DAPA regulates chondrocyte homeostasis through other mechanisms. In our study, by constructing IL-1β-induced human chondrocyte degeneration models and in vitro cultured cartilage explants from OA patients, we observed that DAPA effectively regulated chondrocyte metabolic homeostasis. Additionally, in vivo results showed that DAPA treatment significantly improved the degenerative pathological phenotype in a mouse model of OA, further proving its protective effect on OA development. We further discovered that DAPA promoted ECM synthesis and inhibited ECM degradation in chondrocytes. We combined the transcriptomic and proteomic data from DAPA-treated samples to explore the mechanism underlying this dual protective effect. Using specific inhibitors of each pathway, we identified signaling pathways closely related to OA. Subsequent target protein intervention experiments revealed that DAPA exerts its effects through two pathways: (1) it activates the AMPK signaling pathway by directly binding to AMPKα, and (2) it inhibits the MAPK signaling pathway and relieves the suppression of AMPK signaling by SGLT2 by targeting SGLT2 in degenerative chondrocytes. Ultimately, this leads to enhanced autophagy via the inhibition of mTORC1 activity, thereby reducing chondrocyte damage.

The widely recognized biological activity of DAPA stems from its ability to bind to the membrane protein SGLT2. Our research revealed that SGLT2 expression was significantly elevated in the cartilage of patients with OA and the joint cartilage of mice with DMM-induced OA. Additionally, increased SGLT2 expression was observed in IL-1β-treated chondrocytes in vitro, suggesting that SGLT2 plays an important role in cartilage degeneration. Degenerated chondrocytes upregulate SGLT2 to enhance glucose uptake; however, this also exacerbates cellular damage. In vitro studies have shown that SGLT2 overexpression leads to metabolic imbalance and degeneration of chondrocytes by mediating inflammation and impaired autophagy. Chronic low-grade inflammation is a key driver of OA progression, promoting cartilage degradation by upregulating catabolic enzymes (e.g., ADAMTS and MMP families) and inhibiting anabolic factors. DAPA delays OA progression by activating AMPK signaling and inhibiting MAPK signaling, thereby reducing the release of inflammatory mediators and inflammation. Autophagy is crucial for maintaining cartilage homeostasis and preventing stress-induced damage. Although previous studies have shown that DAPA enhances autophagic flux to sustain cellular homeostasis, its exact mechanism of action in chondrocytes remains unclear. Our study confirmed that the autophagy-enhancing effect of DAPA is closely related to mTORC1 activity. Mechanistically, SGLT2 overexpression impaired AMPK-mediated protective effects and activated MAPK-mediated chondrocyte injury. Given that AMPK activation is a key driver of mTORC1 inhibition and autophagy induction, we demonstrated through pathway activator experiments that AMPK activation counteracted MAPK activation and increased mTORC1 activity induced by SGLT2 overexpression, thereby improving chondrocyte metabolism. Similarly, treatment with p38 and JNK1/2 inhibitors ameliorated SGLT2-induced AMPK suppression and mTORC1 activation. These results indicated functional antagonistic crosstalk between the AMPK and SGLT2/MAPK pathways in degenerative chondrocytes, converging at mTORC1 to jointly regulate chondrocyte autophagy. While elucidating the key target mechanism of DAPA’s protective effect, we found that SGLT2 knockdown did not significantly affect the ability of DAPA to promote ECM synthesis, suggesting that this effect may be independent of SGLT2 or may involve other unidentified targets.

While elucidating the key target mechanism through which DAPA exerts its protective effects, we found that knocking down the expression of SGLT2 in chondrocytes did not significantly alter the ability of DAPA to promote the synthesis of extracellular matrix (ECM) in chondrocytes. This suggests that the promotion of ECM synthesis by DAPA may be independent of SGLT2 or may involve other unidentified targets. We utilized a combination of DARTS, proteomics analysis, CETSA, SPR, and MST technologies to systematically identify the potential target mechanisms, and for the first time, identified AMPKα as a novel binding target of DAPA. Further molecular dynamics simulations and DARTS-western blot experiments using truncated AMPKα domains confirmed that DAPA directly binds to the kinase domain of AMPKα. In-depth mechanistic studies revealed that DAPA does not alter the AMP:ATP or ADP:ATP ratio in cells, and knockdown of upstream kinases LKB1 and CaMKKβ did not affect the ability of DAPA to activate AMPK. Combined with in vitro AMPK kinase activity assays, these results provided the first evidence that DAPA directly enhances AMPK activity and activates the AMPK signaling pathway through direct targeting of the AMPKα kinase domain. This activation mechanism is significantly different from that of known pharmacological activators (such as A-769662, MK-8722, and salicylates), which typically activate AMPK by binding to allosteric drug and metabolite (ADaM) sites. Therefore, DAPA is a novel direct activator of AMPK and its binding mechanism offers new avenues for the development of AMPK activators that directly target the kinase domain. Finally, functional experiments confirmed that knocking down AMPKα expression in chondrocytes completely blocked DAPA’s ability to promote ECM synthesis, confirming that AMPKα is the key downstream target through which DAPA plays a central role in maintaining chondrocyte metabolic homeostasis.

AMP-activated protein kinase (AMPK) is a heterotrimeric complex composed of a catalytic α subunit and regulatory β and γ subunits. Each subunit is encoded by multiple genes, leading to various isoform combinations. AMPK is universally recognized as a central regulator sensing changes in intracellular ATP levels and maintaining cellular energy homeostasis, playing a pivotal hub role in cellular energy metabolism and stress responses [38–40]. However, substantial evidence indicates that aberrant AMPK signaling is closely linked to the onset and progression of OA [41, 42]. Specifically, compared to normal tissues, both OA patients and multiple OA model mice exhibit significantly reduced phosphorylation levels at the key activation site (Thr172) of the AMPKα subunit, alongside a significant downregulation of overall AMPK expression in articular cartilage [25, 26, 43, 44]. Genetic evidence further reveals that specific deletion of the AMPKα1 and α2 subunits in cartilage accelerates post-traumatic OA progression and exacerbates joint damage in mice [45]. Collectively, these findings establish AMPK activation as a crucial protective factor for articular cartilage. Consequently, restoring AMPK activity to mitigate inflammation and slow OA progression has emerged as a promising novel therapeutic strategy. In this context, this study identified the anti-diabetic drug DAPA as an AMPK-targeted activator, confirming AMPKα as its direct target. DAPA directly binds to and activates AMPKα (while independently antagonizing SGLT2 function). Acting as an upstream driver, the activated AMPKα plays a key role in orchestrating the energy, metabolic, and inflammatory homeostasis of chondrocytes. Crucially positioned at the core, AMPKα activation is not only a central switch regulating the balance between catabolism and anabolism in chondrocytes, but this study also reveals that it mediates DAPA’s core protective effect against SGLT2 hyperactivation-induced autophagic impairment: AMPK activation promotes the restoration of autophagic flux through downstream mechanisms. Therefore, AMPKα is not only the direct target of DAPA, but also the upstream hub coordinating its multiple downstream protective effects, including restoration of autophagy and inhibition of MAPK signaling. The elucidation of DAPA’s mechanism of action in this study complements the potential therapeutic value of other AMPK activation strategies in OA. Experimental studies show that the selective AMPK agonist AICAR significantly inhibits the catabolic response in chondrocytes induced by inflammatory cytokines [41]. Metformin delays OA progression by upregulating AMPKα1 and phosphorylated AMPKα1 expression in mouse and rat joint cartilage and effectively alleviates pain sensitivity induced by DMM surgery by regulating AMPKα1 expression in dorsal root ganglion (DRG) cells [25]. These findings further reveal the potential protective role of AMPK in OA pathology and provide an important theoretical basis for developing OA treatment strategies based on AMPK signaling regulation.

From biological and functional perspectives, DAPA plays an important role in OA treatment by inhibiting inflammation and regulating autophagy. Inflammation plays a crucial role in OA progression by upregulating the expression of catabolic enzymes (such as ADAMTS and MMP families) and inhibiting the expression of anabolic factors, leading to cartilage degeneration. DAPA targets and binds to AMPKα and SGLT2 to reduce the release of inflammatory mediators and inhibit inflammation, thereby slowing OA progression. Moreover, autophagy plays a key role in OA pathogenesis by mitigating various stress responses during chondrocyte damage, thereby maintaining cartilage homeostasis and providing protection [46]. Previous studies have shown that DAPA enhances the autophagic flux, thereby maintaining cellular homeostasis [47]. However, the specific regulatory mechanisms of DAPA in autophagy during chondrocyte damage remain unclear. Our study showed that DAPA-mediated autophagy enhancement was closely associated with reduced mTOR activity. AMPK, a key kinase involved in autophagy induction, effectively promotes the activation of activated [48, 49]. Our research further revealed that DAPA activated AMPK in a dose-dependent manner, thus inhibiting mTORC1 and upregulating the expression of the autophagy marker LC3 in chondrocytes. In addition, in the degenerative chondrocyte model, we found that DAPA targets SGLT2, which inhibits the abnormal catabolism of chondrocytes and enhances autophagy activity, further supporting its potential protective role in OA treatment. We used CRISPR/Cas9 technology to create an SGLT2 knockout (SGLT2 KO) chondrocyte line and used SGLT2 overexpression plasmids to investigate the relationship between SGLT2 and autophagy, as well as the underlying molecular mechanisms. These results indicated that SGLT2 inhibition significantly increased autophagy levels in chondrocytes, thereby alleviating chondrocyte damage.

This study confirmed that DAPA is an effective stimulator of chondrocyte proliferation and revealed a new strategy for enhancing chondrocyte function and maintaining cartilage integrity. Therefore, DAPA holds promise as a potential therapeutic agent for diseases and conditions associated with chondrocyte proliferation, particularly osteoarthritis. Our research further clarified that the regulatory effect of DAPA on chondrocytes is primarily mediated by AMPKα and SGLT2. This provided strong support for DAPA as an OA treatment drug and offered new insights into the role of AMPK and SGLT2 in chondrocyte function and OA development. More importantly, this study identified SGLT2 as a new target of DAPA, advancing our understanding of drug mechanisms and potential interactions with AMPK or SGLT2. We are optimistic that, as an SGLT2 inhibitor, DAPA demonstrates significant cartilage-protective effects and is expected to bring substantial therapeutic benefits to patients with OA. Based on the promising results of preliminary studies, large-scale clinical trials in patients with OA will provide further evidence of the drug’s efficacy.

However, we acknowledge the limitations of this study. Systemic administration of DAPA may affect the entire joint structure and various pathological phenotypes. However, this study focused only on the effects of DAPA on cartilage tissue and subchondral bone, excluding the impact on synovial inflammation, pain phenotype, and particularly on the potential effects on AMPKα expression in DRG cells [25, 45]. As an antidiabetic drug, DAPA may also lead to weight loss in patients with obesity, which could indirectly improve the negative impact of obesity on OA [50], potentially benefiting long-term knee joint prognosis in obese patients, although this aspect was not explored in depth in this study. Furthermore, other in vivo characteristics and pharmacokinetic assessments of the drug for OA treatment (including optimal dosage, dosing frequency, administration route, and treatment duration) need to be explored in primate models and human clinical trials. In target research, although we were the first to confirm the direct binding of DAPA to AMPKα, the specific binding sites with AMPKα1 and AMPKα2 have yet to be thoroughly explored. Furthermore, precisely locating DAPA’s binding site on AMPKα1/α2 and systematically evaluating its binding affinity to targeted mutants is crucial for confirming binding specificity and provides a structural basis for comprehensively elucidating DAPA’s mechanism of action. Although such site-directed mutagenesis studies are not addressed in this work, they form the central focus of our subsequent research, dedicated to elucidating the structural mechanism of the DAPA-AMPKα interaction.

Overall, the functional studies, including in vitro and in vivo experiments, support the feasibility of using DAPA as a novel treatment option for patients with OA in clinical practice. Moreover, through a combination of different methods, this study confirms AMPKα as a new target of DAPA and clarifies the important roles of AMPKα and SGLT2 in its cartilage-protective effects. (Graphical abstract). Therefore, this study provides new insights into the mechanisms of action of DAPA and strong evidence for expanding its clinical applications.

## Materials and Methods

### Cell cultures and OA patients cartilage explants culture

All procedure has been approved by the Ethics Committee of Qilu Hospital of Shandong University and all patients have signed informed consent forms. Detailed patient information is shown in Table 1.

**Table 1:** Characteristics details of the patients enrolled in the study.

Case no.	Age (years)	Gender	Diagnosis	Surgical Procedures
Case 1	32	M	Cartilage damage	Knee arthroscopy
Case 2	37	F	Cartilage damage	Knee arthroscopy
Case 3	28	M	Cartilage damage	Knee arthroscopy
Case 4	26	F	Cartilage damage	Knee arthroscopy
Case 5	27	F	Cartilage damage	Knee arthroscopy
Case 6	19	F	Cartilage damage	Knee arthroscopy
Case 7	27	F	Cartilage damage	Knee arthroscopy
Case 8	25	M	Cartilage damage	Knee arthroscopy
Case 9	29	F	Cartilage damage	Knee arthroscopy
Case 10	27	F	Cartilage damage	Knee arthroscopy
Case 11	67	F	Knee osteoarthritis	Total knee replacement
Case 12	65	M	Knee osteoarthritis	Total knee replacement
Case 13	53	F	Knee osteoarthritis	Unicondylar replacement
Case 14	55	F	Knee osteoarthritis	Unicondylar replacement
Case 15	67	M	Knee osteoarthritis	Total knee replacement
Case 16	62	M	Knee osteoarthritis	Unicondylar replacement
Case 17	58	M	Knee osteoarthritis	Unicondylar replacement
Case 18	72	M	Knee osteoarthritis	Total knee replacement
Case 19	69	F	Knee osteoarthritis	Total knee replacement
Case 20	67	F	Knee osteoarthritis	Total knee replacement

Human normal articular chondrocytes were harvested from knee trauma patients who underwent knee arthroscopy surgery. After washing with PBS containing 1% penicillin-streptomycin (Solarbio; P1400), the tissues were digested with 0.25% trypsin-EDTA solution (Solarbio; T1350) for 20 min, followed by low-speed centrifugation (1000 rpm, 20 °C) for 3 min. The excess trypsin was discarded. Articular tissue fragments were resuspended in collagenase Ⅱ (Solarbio; C8150) and incubated at 37 °C in a cell incubator for 2–4 h until completely digested. Digested tissues were filtered aseptically using a 70 μm cell filter, and the filtrates were centrifuged at low speed (1000 rpm, 20 °C) for 5 min. The supernatants were discarded and the cell precipitates were resuspended in DMEM/F-12 medium (Gibco-BRL; A4192001) containing 10% fetal bovine serum (Gibco-BRL; 30044333) and 1% penicillin-streptomycin. The extracted chondrocytes were collected and seeded into six-well plates for subsequent studies and cultured at 37 °C in a humidified incubator (5% CO₂).

Cartilage explants ( ~ 4 x 4 mm x full-thickness) were collected from the femoral condyles of OA patients who underwent surgery for osteoarthritis of the knee joint. After thorough washing and weighing, explants were cultured in 6-well plates (one explant per well in 2 mL of medium) containing F12/DMEM supplemented with 10% FBS and 1% penicillin/streptomycin. Following a 12-hour acclimatization period, the explants were subjected to treatment with vehicle control, 10 μM, or 20 μM DAPA. The culture medium was entirely replaced with fresh medium containing the corresponding treatments every 48 h to maintain consistent drug concentration and nutrient supply. This treatment regimen continued for 7 days, after which the explants were processed for histological analysis (fixed in 4% paraformaldehyde, decalcified if necessary, paraffin-embedded, and sectioned for immunohistochemistry) [51].

### CRISP-Cas9 knockout of SGLT2, LKB1, and CAMKK2

The gene sequence of the sgRNA targeting SGLT2 is: 5′­CACTGTGGGC-GGCTACTTCC-3′. The gene sequence of the sgRNA targeting LKB1 is: 5′-AGCTT GGCCC GCTTG CGGCG-3′. The gene sequence of the sgRNA targeting CAMKK2 is: 5′­TGGAAGGTTTGATGTCACGGTGG-3′. This designed sequence was inserted into an sgRNA vector containing the spCas9 gene and the puromycin resistance gene. The CRISPR/Cas9 single-vector lentivirus was used to deliver the Cas9 protein and sgRNA expression cassette into actively growing C28/I2 cells. Ultimately, the SGLT2 KO C28/I2 cell line, LKB1 KO C28/I2 cell line and CAMKK2 KO C28/I2 cell line was successfully generated, and the KO efficiency was measured by Western blot.

### Construction and transfection of overexpression plasmids

The SGLT2 overexpression plasmid was constructed by linking the coding sequence of the human SGLT2 gene to pDNA3.1. The non-targeting vector was used as a negative control. The SGLT2-KO-C28/I2 cell line and human primary chondrocytes were transiently transfected using Lipofectamine 8000 reagent (Beyotime, China) according to the manufacturer’s instructions.

### Construction and transfection of Small interfering RNA

AMPKα and/or SGLT2 siRNAs were transfected into human chondrocytes using Lipofectamine 8000 transfection reagent (Beyotime, China). After 24 h of transfection, DAPA and/or IL-1β were added for stimulation. The chondrocytes were then collected for subsequent experiments.

### Total RNA extraction, reverse transcription, and quantitative real-time PCR

All procedures were carried out by the manufacturer’s protocol, total RNA from cells or tissues was extracted using TRIzol reagent (Thermo Fisher, USA), and cDNA synthesis was performed using a real-time PCR Master Mix (Toyota, Japan). SYBR Green PCR Master Mix (Toyobo, Japan) was used for qRT-PCR reactions. Target gene mRNA expression was calculated using the ΔΔCT method, and fold changes of mRNA levels were normalized to the housekeeping gene. Each experiment was repeated three times. The following primer sequences were used in this study are listed in Table 2.

**Table 2:** Primers used for quantitative real-time PCR.

Target	Forward primers, 5′–3′	Reverse primers, 5′–3′
COX2(Human)	TCCTTGGGTGTCAAAGGTAAA	TGGCCCTCGTTATGATCTG
iNOS(Human)	CGTGGAGACGGGAAAGAAGT	GACCCCAGGCAAGATTTGGA
MMP13 (Human)	ATTAAGGAGCATGGCGACTTCT	GCCCAGGAGGAAAAGCATGA
ADAMTs4(Human)	ATGGCTATGGGCACTGTCTC	CTGGCGGTCAGCATCATAGT
Aggrecan(Human)	AAACCTGGCGTGAGAACTGT	CCACTGACACACCTCGGAAG
COL2(Human)	GATGGCTGCACGAAACATACC	GCCCTATGTCCACACCGAAT
COMP(Human)	GATCACGTTCCTGAAAAACACG	GCTCTCCGTCTGGATGCAG
β-actin(Human)	CATGTACGTTGCTATCCAGGC	CTCCTTAATGTCACGCACGAT
NFATc1(Mouse)	CATCCTGTCCAACACCAAAGTC	GTGTTCTTCCTCCCGATGTCTG
c-FOS(Mouse)	AGTAGAGCAGCTATCTCCT	AACGCAGACTTCTCATCTT
ACP5(Mouse)	CAGCAGCCAAGGAGGACTAC	CACATAGCCCACACCGTTCTC
CTSK(Mouse)	ATGGAAGAAGACTCACCAGAAG	CCACTTCTTCACTGGTCATGTC
MMP9(Mouse)	GGACCCGAAGCGGACATTG	CGTCGTCGAAATGGGCATCT
β-actin(Mouse)	GGCTGTATTCCCCTCCATCG	CCAGTTGGTAACAATGCCATGT

### Western Blot analysis

For Western blotting, protein samples from cells or tissues were separated by SDS-PAGE and transferred onto a polyvinylidene fluoride (PVDF) membrane. The membrane was blocked with 5% non-fat milk (P0216, Beyotime) at room temperature for 2 h. It was then incubated with the specified primary antibody at 4 °C for 16 h, followed by incubation with the appropriate secondary antibody at room temperature for 2 h. Protein expression was detected using ECL chemiluminescent solution (MA0186, Meilunbio), and the images were visualized using the Tanon imaging system (Shanghai, China). Finally, the band intensity was quantified using Image J software, and normalized with β-actin as the internal reference after background subtraction (Table 3).

**Table 3:** Antibody information.

Antibody	Company	Catalog #	Application/Dilution
Collagen II	Abclonal	A19308	WB (1:1000); IHC (1:100)
Aggrecan	Abclonal	A11691	WB (1:1000); IF (1:100)
ADAMTS4	Proteintech	11865-1-AP	WB (1:1000)
MMP13	Proteintech	18165-1-AP	WB (1:1000); IHC (1:100); IF (1:100)
COX2	Proteintech	66351-1-Ig	WB (1:1000); IHC (1:100); IF (1:100)
iNOS	Proteintech	22226-1-AP	WB (1:1000)
p38	CST	8690	WB (1:1000)
p-p38	CST	4511	WB (1:1000)
JNK	CST	9252	WB (1:1000)
p-JNK	CST	9255	WB (1:1000)
β-Actin	ABclonal	AC038	WB (1:1000)
p62	ABclonal	A19700	WB (1:1000)
LC3B	CST	3868	WB (1:1000)
SGLT2	Invitrogen	PA5-101893	WB (1:1000); IHC (1:100)
SIRT1	Abcam	ab110304	WB (1:1000)
NFATc1	ABclonal	A24872	WB (1:1000); IF (1:100)
Cathepsin K	Proteintech	11239-1-AP	WB (1:1000)
MMP-9	Proteintech	10375-2-AP	WB (1:1000)
ACP5	Proteintech	11594-1-AP	WB (1:1000)
c-Fos	Invitrogen	MA5-15055	WB (1:1000)
PINK1	ABclonal	A7131	WB (1:1000)
Parkin	ABclonal	A11172	WB (1:1000)
mTOR	Proteintech	81670-1-RR	WB (1:1000)
p-mTOR	Proteintech	80596-1-RR	WB (1:1000)
eIF4B	Proteintech	17917-1-AP	WB (1:1000)
p-eIF4B	Proteintech	80059-1-RR	WB (1:1000)
p70S6K	Proteintech	14485-1-AP	WB (1:1000)
p-p70S6K	Proteintech	81592-1-RR	WB (1:1000)
RPS6	Proteintech	80208-1-RR	WB (1:1000)
p-RPS6	Proteintech	29223-1-AP	WB (1:1000)
AMPKα	CST	2532	WB (1:1000)
p-AMPKα1/α2	CST	4185	WB (1:1000)
AMPKα1	CST	2795	WB (1:1000)
AMPKα2	CST	2757	WB (1:1000)
AMPKγ1	CST	4187	WB (1:1000)
AMPKγ2	CST	2536	WB (1:1000)
AMPKγ3	CST	2550	WB (1:1000)
AMPKβ1	Abcam	ab32112	WB (1:1000)
AMPKβ2	Abcam	ab277780	WB (1:1000)
LKB1	CST	3047	WB (1:1000)
CAMKK2	CST	16810	WB (1:1000)
p-ACC	CST	11818	WB (1:1000)
ACC	CST	3676	WB (1:1000)
p-TSC2	CST	5584	WB (1:1000)
TSC2	CST	4308	WB (1:1000)
p-RPTOR	CST	2083	WB (1:1000)
RPTOR	CST	48648	WB (1:1000)
His Tag	Immunoway	YM8314	WB (1:1000)

### Enzyme-linked immunosorbent assay

Human COMP ELISA Kit (RK09148), Human IL-6 ELISA Kit (RK00004) and Human IL-1β ELISA Kit (RK00001) were purchased from ABclonal (Wuhan, China). The optical density value of each well was measured at a wavelength of 450 nm using a FlexStation 3 multi-mode microplate reader (Molecular Devices). Concentrations for each sample were calculated, and data analysis was performed using GraphPad Prism 10.3.1 software.

### Immunofluorescence

The medium from the cell culture plates was discarded and human chondrocytes were washed using PBS. Cells were fixed in cell culture plates using 4% paraformaldehyde for 20–30 min and permeabilized with 0.2% Triton X-100 for 2–5 min. After washing with PBS, the cells were treated with 5% bovine serum albumin for 30 min at room temperature. Specific antibodies were added, followed by overnight incubation at 4 °C. Next, rhodamine-labeled goat-anti-rabbit IgG (Beijing Golden Bridge Biotechnology, ZF-0316) was added and incubated at room temperature away from light for 1 h. After four subsequent 5 min PBS washes, cells were optionally counterstained with DAPI for nuclear visualization and washed again (3x PBS). Coverslips were mounted using anti-fade mounting medium, or cells were imaged directly in PBS. Images were acquired using a laser scanning confocal microscope (LSM900, Zeiss, Germany) and fluorescence intensity was quantified using Image J.

### Alcian Blue Staining

The chondrocytes were seeded in a 6-well culture plate and treated with IL1β and/or different concentrations of DAPA for different time point, with media changes every two days. After the incubation period, the cells were stained with Alcian Blue staining solution (G2541, Solarbio, Beijing, China) to analyze the content of acidic glycosaminoglycans.

### Quantification of ATP, ADP, and AMP

In this study, ATP, ADP, and AMP were quantitatively analyzed using the Solarbio BC5114 kit (Catalog No.: BC5114) in combination with high-performance liquid chromatography (HPLC) on a Dionex UltiMate 3000 system. The experiment strictly followed the standardized operating procedures provided by the kit manufacturer.

### Metabolic Stress Control Experiment

This experiment established a metabolic stress model using human chondrocytes, and AMPK phosphorylation (Thr172) was detected by Western blotting. The cells were pre-synchronized with serum-free treatment for 12 h. The experimental groups included: control group (no treatment), DAPA group (10 μM), oligomycin group (10 μM), 2-DG group (10 mM), DAPA + oligomycin group (oligomycin pre-treatment followed by DAPA co-treatment), and DAPA + 2-DG group (2-DG pre-treatment followed by DAPA co-treatment), with three independent experimental repeats. After treatment, total cell protein was extracted, and Western blotting was used to detect the expression of p-AMPKα and total AMPKα antibodies. ATP/AMP levels were measured using a commercial kit (Solarbio; BC5114) and high-performance liquid chromatography (HPLC) to determine the intracellular ATP and AMP concentrations, and the ATP/AMP ratio was calculated to assess the energy status.

### In Vitro AMPK Kinase Assay

To definitively determine whether DAPA directly activates AMPK, an in vitro kinase assay was performed using purified recombinant human AMPK heterotrimeric complex (α1β1γ1). Briefly, 100 ng of AMPK protein was pre-incubated with increasing concentrations of DAPA (10 µM) or equivalent volumes of solvent control (DMSO) in reaction buffer (40 mM HEPES pH 7.4, 80 mM NaCl, 5 mM MgCl₂) for 15 min at 30 °C. Reactions were initiated by adding 200 µM ATP and incubated for 30 min at 30 °C. Positive controls included the AMP, while negative controls omitted ATP. Reactions were terminated by adding 5× Loading SDS-PAGE sample buffer and boiling at 95 °C for 10 min. Proteins were resolved on 10% SDS-PAGE gels, transferred to PVDF membranes, and probed with primary antibodies against phospho-AMPKα (Thr172) and total AMPKα. Band intensity was quantified using ImageJ software (NIH), and phospho-AMPKα (Thr172) levels were normalized to total AMPKα to calculate relative activation fold-change over vehicle controls. All experiments included ≥3 technical replicates and were independently repeated three times.

### DMM surgery-induced murine OA

In this study, mice were housed in a specific pathogen-free environment with controlled temperature (20 °C), humidity (40-70%), and a 12-hour light/dark cycle. Food and water were provided ad libitum. At the end of the experiment, all mice were humanely euthanized, and cartilage samples were collected for subsequent analyses. The entire experimental procedure was conducted using a blinded method. All animal experiments complied with institutional guidelines for animal care and use and were approved by the Ethics Committee of Qilu Hospital, Shandong University. Twelve-week-old male C57BL/6 J wild-type (WT) mice were used, and OA was surgically induced via the destabilization of the medial meniscus (DMM) model [52]. In this model, male mice exhibit greater susceptibility to post-traumatic OA compared to females, potentially due to the chondroprotective effects of estrogen in females and the exacerbating role of male hormones in cartilage damage. Prior to surgery, mice were anesthetized, and the surgical site was shaved and disinfected. The right knee joint was exposed through a medial parapatellar approach, with the patella laterally dislocated and the knee fully flexed. The anterior medial meniscotibial ligament was transected using a microsurgical blade. Subsequently, the medial meniscus was manually displaced with fine forceps to confirm complete ligament rupture. The joint cavity was irrigated with saline, and the wound was sutured and disinfected. A sham surgery group served as the control, undergoing identical procedures except for the transection of the medial meniscotibial ligament. All mice were euthanized 12 weeks post-surgery. Starting on day 3 after surgery, OA mice received daily oral administration of Dapagliflozin at doses of 1.0 mg/kg or 10.0 mg/kg.

### Micro-CT, histology and Immunohistochemistry (IHC)

At 12 weeks post-surgery, mouse knee joints were harvested, fixed in 4% paraformaldehyde for 24 hours, and stored in 70% ethanol prior to Micro-CT scanning. Scanning was performed using a small-animal computed tomography system (Quantum GX2, PerkinElmer, USA) with parameters set to 90 kV and 88 mA. Following three-dimensional reconstruction and dataset orientation, osteophyte development was quantified using the built-in 3D analysis software (CT Analyzer, USA). Total osteophyte number was measured by manually segmenting a region of interest (ROI) encompassing osteophytic tissue, characterized by its deviation from the cortical bone boundary.

Following imaging, joints were decalcified in EDTA decalcification solution for 3 weeks. Decalcified tissues were paraffin-embedded and sectioned into 5 μm-thick slices using a rotary microtome (Leica RM2235, Germany). Tissue sections were stained using commercial H&E and Safranin O-Fast Green staining kits (Servicebio, Wuhan, China) according to the manufacturer’s protocols. Two blinded investigators quantified chondrocyte density, cartilage thickness, and subchondral bone plate (SBP) thickness. Osteoarthritis severity was graded using the Osteoarthritis Research Society International (OARSI) scoring system.

For IHC staining, deparaffinized and rehydrated sections undergo antigen retrieval, blocking of endogenous peroxidase, and are then incubated with primary antibodies overnight at 4 °C. Sections were then incubated with HRP-conjugated secondary antibodies for 60 minutes at room temperature. Positive signals were visualized using 3,3′-diaminobenzidine (DAB; ZSGB-BIO, China), and nuclei were counterstained with hematoxylin. Images were acquired using a Zeiss microscope (Carl Zeiss, Germany) and quantified with Image J software (NIH, USA). Specifically, color deconvolution separated DAB (brown) and hematoxylin (blue) channels, followed by background subtraction on the DAB channel; positive areas were thresholded using the Huang algorithm, after which articular cartilage regions of interest (ROI) were outlined to calculate the percentage of DAB-positive area, while Integrated Optical Density (IOD) was measured with background normalization; data from ≥5 fields per sample were averaged as a single biological replicate, with all analyses conducted blinded to experimental groups.

### Blood glucose and body weight

Body weight was measured at designated time points using a calibrated electronic balance, with absolute values reported in grams (g). Blood glucose levels were monitored under 6-hour fasting conditions; small blood samples were collected via sterile tail vein puncture and immediately analyzed using a validated and pre-calibrated handheld glucometer with compatible test strips. All procedures were conducted in accordance with approved institutional animal care protocols.

### Bone marrow-derived macrophage extraction, osteoclastogenesis

Bone marrow cells were isolated from the femurs and tibiae of 6- to 8-week-old C57BL/6 J mice under aseptic conditions. The marrow cavity was flushed with complete medium (α-MEM supplemented with 10% FBS and 1% penicillin/streptomycin), and cells were resuspended in complete medium for 24 h. For osteoclastogenesis, non-adherent cells were cultured with 20 ng/mL macrophage colony-stimulating factor (M-CSF; PeproTech, USA) for 6 days, followed by differentiation in medium containing 20 ng/mL M-CSF and 100 ng/mL receptor activator of nuclear factor-κB ligand (RANKL; PeproTech, USA) for an additional 7 days.

### NFATC1 Nuclear Translocalization Assay

BMDMs were pretreated with or without Dapagliflozin overnight, then stimulated with RANKL (100 ng/mL) for 6 h. Immunofluorescence staining was performed to assess NFATC1 nuclear translocation using anti-NFATC1 antibody. Nuclei were counterstained with DAPI, and images were captured using laser scanning confocal microscope (LSM900, Zeiss, Germany).

### TRAP Staining

Tartrate-resistant acid phosphatase (TRAP) staining was conducted on cell or tissues using a commercial kit (G1050-50T; Servicebio, Wuhan, China) according to the manufacturer’s protocol. TRAP-positive cells were visualized under an optical microscope, and multinucleated cells ( ≥ 3 nuclei) were quantified as mature osteoclasts.

### RNA-seq analysis

Chondrocytes from different treatment groups were collected and immersed in TRIzol reagent (Thermo Fisher Scientific, USA), followed by rapid freezing and storage in liquid nitrogen. RNA sequencing (RNA-seq) was performed by LC-Bio Technology (Hangzhou, China). The raw RNA-seq data have been deposited in the NCBI Sequence Read Archive (SRA) under the Accession ID: PRJNA1330511, and are publicly accessible at: https://www.ncbi.nlm.nih.gov/sra/PRJNA1330511. DEGs were identified using the following thresholds: |Fold Change (FC) | ≥ 1.5 and adjusted *p*-value < 0.05. This study employed three stringent statistical criteria for KEGG pathway analysis of transcriptomic data: ① |NES | >1; ② NOM p-val < 0.05; ③ FDR q-val < 0.25. Only gene sets meeting all the above criteria were included for subsequent biological validation. Chord diagrams were generated using the online platform OmicStudio (https://www.omicstudio.cn/home) for data analysis and visualization.

### DARTS, coomassie brilliant blue, and mass spectrometry analysis

The rat chondrocytes were incubated with M-PER lysis buffer (Thermo, USA) at 4 °C, and the cell lysates were collected and centrifuged to remove cell debris. The protein concentration was then measured. Subsequently, the cell lysates were incubated with DMSO or 10 μM dapagliflozin at room temperature for 2 h. Afterward, each sample was mixed with protease (Sigma) and digested at 37 °C for 15 min. Upon completion of digestion, a protease inhibitor mixture was added and the samples were incubated on ice for 10 minutes to stop digestion. Next, 5x SDS sample buffer was added, and the samples were boiled at 95 °C to prepare for analysis. The samples were then loaded onto an SDS-PAGE gel for protein separation, followed by Coomassie Brilliant Blue staining. The target protein bands were excised and digested, then dissolved in 0.1% trifluoroacetic acid, and analyzed by mass spectrometry (Orbitrap Fusion, Thermo). MS data were searched using the internal database Proteome Discoverer against the UniProt target protein database. Furthermore, DARTS experiments were performed on human chondrocytes, and Western blot analysis was conducted to validate the potential target proteins identified by mass spectrometry.

### CETSA

The human chondrocytes were treated with DMSO or DAPA (10 μM) for 1 h at 37 °C and 5% CO₂ in a cell incubator. The cells were then collected and equally divided into multiple sterile Ep tubes. The cell suspensions were digested for 3 min at different temperatures. Samples were lysed by three freeze-thaw cycles, followed by centrifugation at 15,000 × g for 20 min at 4 °C. The supernatant was then collected for Western blot analysis. For the isothermal dose-response experiment, cells were treated with a range of DAPA concentrations (from 0.001 μM to 100 μM) and subjected to thermal denaturation at fixed temperatures. All samples were loaded onto SDS-PAGE gels for subsequent WB analysis to detect target proteins.

### SPR Analysis of AMPKα1/α2 Interaction with DAPA

The AMPKα1 and AMPKα2 proteins were immobilized onto the CM5 sensor chip channels (2 and 4) using the amine coupling method, with 1× PBSP buffer (pH 7.4) as the coupling medium. After activating the chip with an EDC/NHS mixture (10 μL/min for 2 min), ligand proteins diluted to 50 μg/mL in acetate buffer were immobilized at the same flow rate. Subsequently, non-specific binding sites were blocked using ethanolamine. Reference channels (1 and 3) were treated with buffer alone for background subtraction. For interaction analysis, PBSP buffer containing 5% DMSO was used, with solvent correction performed by mixing 4.5 and 5.8% DMSO stock solutions. The analytes were serially diluted in concentration and flowed over the chip at a rate of 30 μL/min for 150 s per cycle. Following binding, the chip was regenerated with 10 mM glycine-HCl (pH 2.0) for 5 min. Kinetic data were globally fitted using the Biacore Insight software with the 1:1 Langmuir model to calculate the dissociation rate constant (kd). Binding curves and associated fitting data were generated using GraphPad Prism 8 software.

### MST Analysis of AMPKα1/α2 Interaction with DAPA

The MicroScale Thermophoresis (MST) analysis for the interaction between AMPKα1/α2 and DAPA was performed as described previously. Recombinant purified AMPKα1 and AMPKα2 proteins were fluorescently labeled in an appropriate buffer using the RED-NHS Protein Labeling Kit. The DAPA stock solution (10 mM in DMSO) was diluted with MST assay buffer to the required working concentrations. The labeled recombinant proteins were co-incubated with serially diluted DAPA in MST reaction buffer. Binding analysis was then conducted using the Monolith NT.115 instrument with the following parameters: 40% Infrared Laser Power and 20% LED power. Data acquisition and processing were performed using NanoTemper analysis software. The dissociation constants (KD) for the interaction between DAPA and AMPKα1/α2 were calculated by fitting the binding data. Binding curves and associated fitting data were generated using GraphPad Prism 8 software.

### Molecular docking simulations

In this study, molecular docking was employed to systematically investigate the interaction between DAPA and AMPKα1 (Q13131) and AMPKα2 (P54646). First, the protein crystal structures were retrieved from the PDB database, and water molecules and non-essential heteroatoms were removed using UCSF Chimera. The atomic charges were then computed using the AMBER14SB force field, and the amino acid protonation states were optimized at physiological pH 7.4 using the H + + 3 online server (http://biophysics.cs.vt.edu/H + +).

The three-dimensional conformation of the ligand DAPA was generated using RDKit and subsequently underwent conformational sampling and energy minimization with the MMFF94 force field. The lowest energy conformation was selected, and AM1-BCC charges were assigned via UCSF Chimera. Flexible docking experiments were performed using AutoDock 4.2, with the docking box centered at coordinates (x = -3.847, y = 2.062, z = 4.077), a grid spacing of 0.375 Å. A global search was conducted using a genetic algorithm, and the top nine binding conformations were output.

Analysis revealed that for AMPKα1 (Q13131), DAPA forms a strong hydrogen bond with ASN155 (3.0 Å), a weaker hydrogen bond with ASP168 (5.1 Å), and interacts hydrophobically with 10 residues, including VAL35 and LEU33 (docking score shown in Table 4). In contrast, for AMPKα2 (P54646), DAPA simultaneously forms hydrogen bonds with GLU143 (2.7 Å) and VAL24 (3.0 Å), while also collaborating with 7 residues, such as VAL24 and LEU22, to form a hydrophobic region (docking score shown in Table 5). Molecular visualization was performed using PyMOL 2.04, and the 2D interaction maps were generated using the Schrödinger Maestro 12.8 academic version.

**Table 4:** AMPKα1(Q13131)-Dapagliflozin Docking score.

Rank	Docking score
pose-1 (kcal/mol)	−8.6
pose-2 (kcal/mol)	−8.6
pose-3 (kcal/mol)	−8.4
pose-4 (kcal/mol)	−8.4
pose-5 (kcal/mol)	−8.0
pose−6 (kcal/mol)	−8.0
pose−7 (kcal/mol)	−7.9
pose−8 (kcal/mol)	−7.7
pose−9 (kcal/mol)	−7.5

**Table 5:** AMPKα2(P54646)−Dapagliflozin Docking score.

Rank	Docking score
pose−1 (kcal/mol)	−7.7
pose−2 (kcal/mol)	−7.4
pose−3 (kcal/mol)	−7.2
pose−4 (kcal/mol)	−7.0
pose−5 (kcal/mol)	−7.0
pose−6 (kcal/mol)	−6.9
pose−7 (kcal/mol)	−6.9
pose−8 (kcal/mol)	−6.8
pose−9 (kcal/mol)	−6.8

### Construction and Transfection of AMPKα1 and AMPKα2 Truncation Mutant Plasmids

AMPKα1 and AMPKα2 truncation mutants were constructed via consecutive deletion of amino acid sequences from the N-r and C-termini, based on their known functional domains. Each plasmid construct incorporated a tag sequence for detection. The specific constructs generated were as follows: For AMPKα1: Full-length (residues 1–536); deletion mutants Δ1 (Δ27–279), Δ2 (Δ302–381), and Δ3 (Δ485–536). For AMPKα2: Full-length (residues 1–521); deletion mutants Δ1 (Δ16–268), Δ2 (Δ291–376), and Δ3 (Δ477–521). C28/I2 cells were transfected with each truncated mutant plasmid for 48 h using Lipofectamine 8000 according to the manufacturer’s protocol.

### Statistical analysis

The sample size (n, referring to the number of independent biological replicates in each experimental group) was determined based on the results of preliminary experiments and previous studies, while taking into account the expected data variability. The n value for each specific analysis is indicated in the corresponding figure legend. During the data collection and analysis phase, researchers implemented blinding for group allocation and randomly assigned experimental subjects to each treatment group. All data analyses were performed using GraphPad Prism (Version 10; GraphPad Software, San Diego, CA, USA). For continuous variables, normality tests and homogeneity of variance tests were first conducted. After verification by the Shapiro-Wilk test and Q-Q plots, if the data conformed to a normal distribution, they were described as mean ± standard deviation (Mean ± SD); otherwise, they were expressed as median and interquartile range (Median (IQR)). Graphs for intergroup comparisons are typically presented with means and 95% confidence intervals (95% CI). Prior to conducting intergroup comparisons, we evaluated the assumptions of parametric tests through the Shapiro-Wilk test (for normality), Levene’s test (for homogeneity of variance), and residual plots. If all assumptions were satisfied: a two-tailed unpaired t-test was used for comparisons between two independent groups; for comparisons involving three or more groups, one-way or two-way analysis of variance (ANOVA) was selected based on the experimental design. If the ANOVA results indicated significant differences, Tukey’s HSD post hoc test was applied for multiple comparisons. If the data seriously violated the assumptions of parametric tests (e.g., non-normal distribution or non-homogeneous variance, and data transformation failed to improve this), non-parametric tests were adopted: the Mann-Whitney U test was used for comparisons between two independent groups; the Kruskal-Wallis H test was used for comparisons involving three or more groups. If the results were significant, Dunn’s post hoc test was employed, with P-value adjustment performed using Holm’s method. Statistical significance was defined as a two-tailed P < 0.05. All specific statistical test methods, and exact *P* values are reported in the corresponding figure legends.

## Supplementary information

Supplementary Figure

Original Western Blots

Data Set

## Data Availability

Data are available from the corresponding author upon reasonable request.
