# Supplementary material for: Dapagliflozin regulates chondrocyte homeostasis and protects against osteoarthritis via targets AMPKα and SGLT2
Source: Cell Death Discov. 2026 Mar 19;12:217. doi: 10.1038/s41420-026-03016-y (PMC13172547; doi:10.1038/s41420-026-03016-y)
Supplement: Supplementary file 1 — Supplementary Figure [file 41420_2026_3016_MOESM1_ESM.docx]

**Supplementary Figure**

**
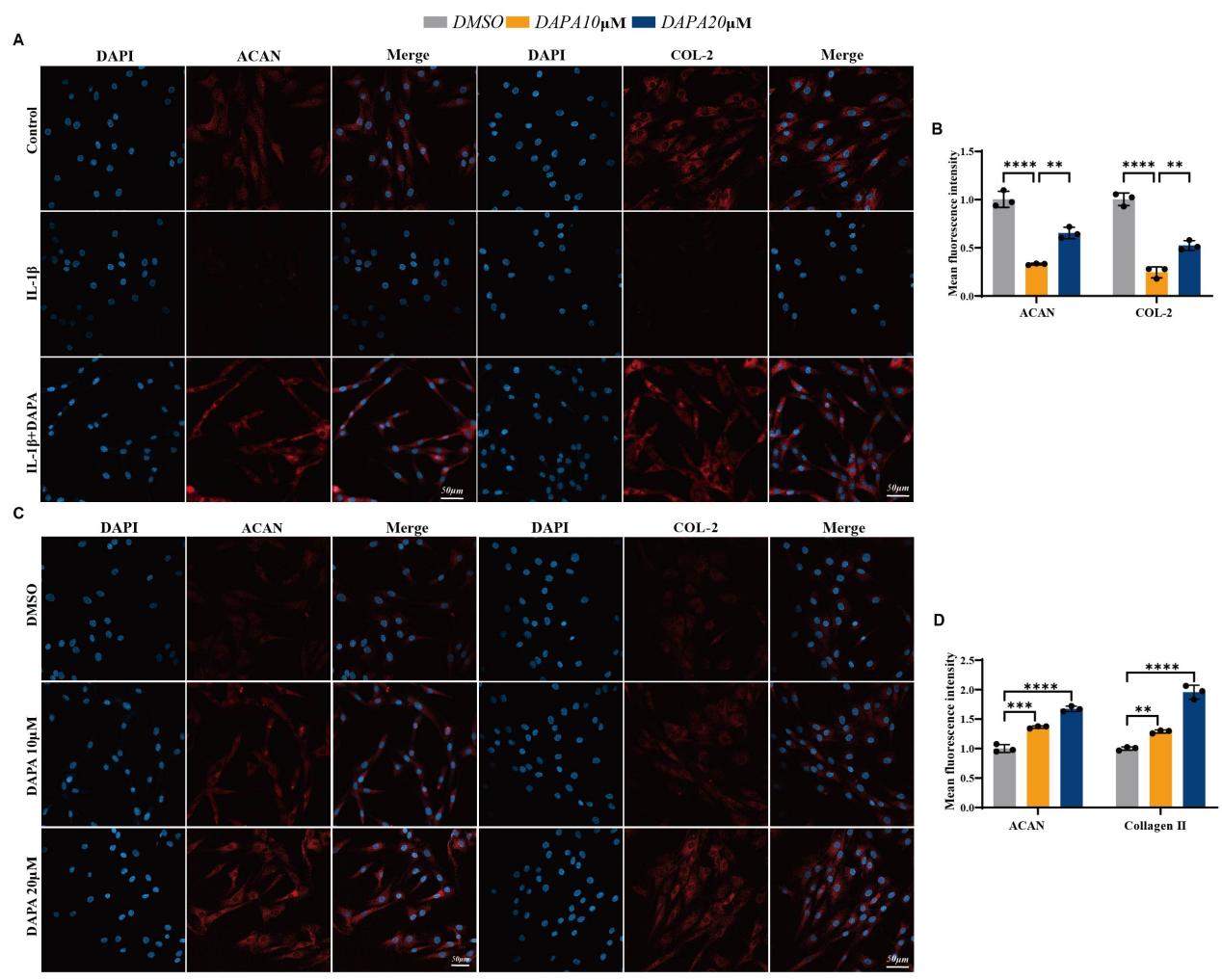
**

**Supplementary Figure 1. DAPA alleviates IL-1β-mediated chondrocyte catabolism and enhances chondrocyte anabolism. (A-B)** Human normal chondrocytes were treated with or without IL-1β and DAPA for 48 h, and analyzed by immunofluorescence staining of ACAN and COL-2. Scale bar=50 µm. Positive staining for ACAN and COL-2 were quantified (n=3). **(C)** Human normal chondrocytes were treated with different concentrations of DAPA for 48 h, and analyzed by immunofluorescence staining of ACAN and COL-2. Scale bar=50 µm. **(D)** Quantification of (C) by Image J (n=3). Statistical analysis was performed using one-way ANOVA with Tukey's post hoc test. Significant differences are indicated as follows: *P < 0.05, and **P < 0.01.

**
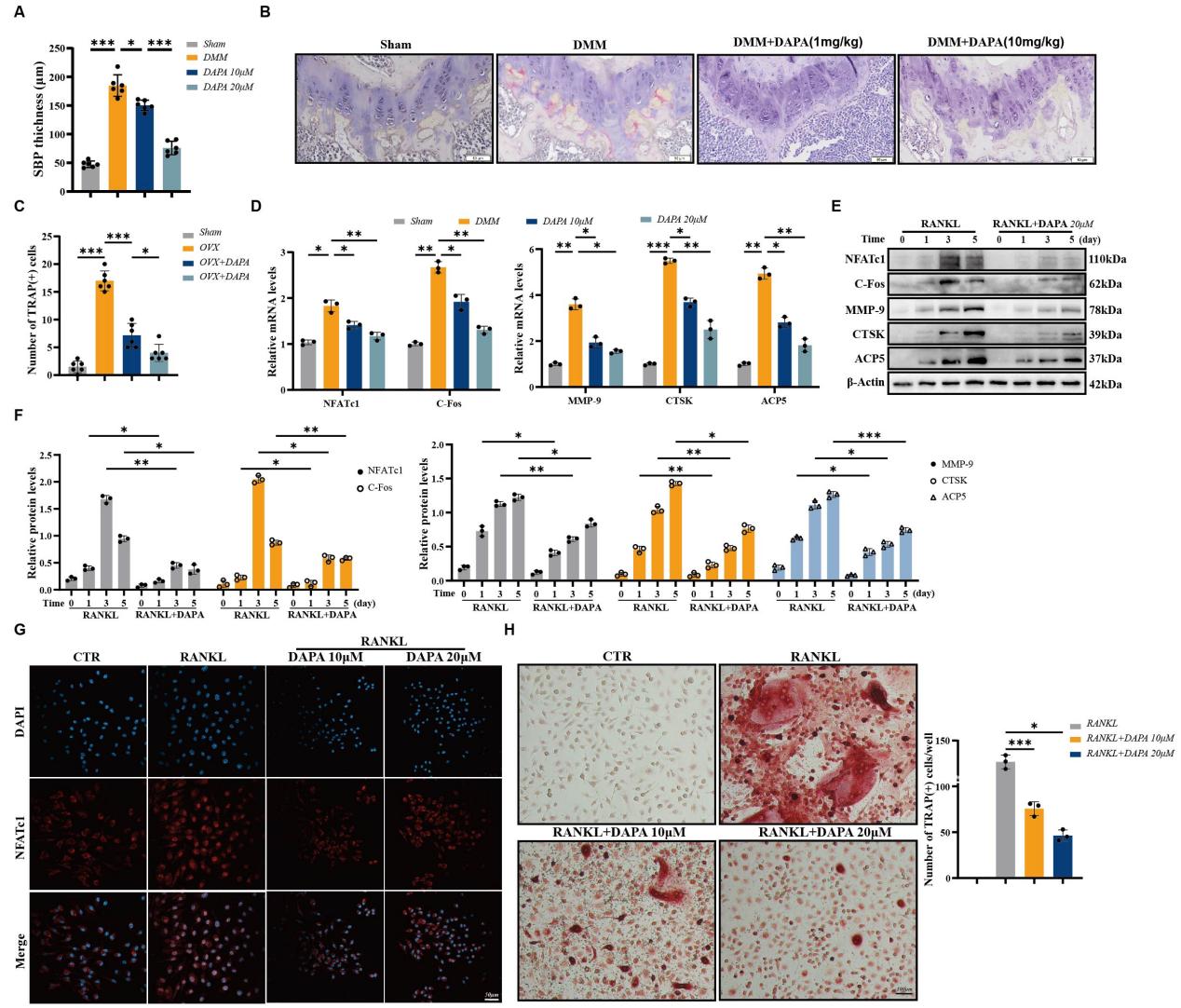
**

**Supplementary Figure 2. DAPA inhibits the abnormal remodeling of subchondral bone by suppressing osteoclastogenesis. (A)** Scoring of HE staining cartilage thickness. **(B)** TRAP staining of subchondral bone in the tibia of mice. **(C)** Quantification of TRAP-positive cell numbers(n = 6/group). **(D)** qRT-PCR analysis of relative mRNA levels of osteoclast-related genes, c-Fos and NFATc1 (n = 3/group). **(E)** BMDM cells were stimulated with RANKL (100 ng/mL) in the presence or absence of DAPA (10 µM) for 0, 1, 3, and 5 days. Western blotting was used to detect the protein levels of c-Fos, NFATc1, MMP9, CTSK, and ACP5. **(F)** Quantification of the Western blot results shown in panel E. **(G)** Immunofluorescence detection of NFATc1 nuclear translocation (scale bar = 50 µm). **(H)** TRAP staining was used to quantify osteoclast numbers. Multinucleated cells (≥3 nuclei) were identified as osteoclasts (scale bar = 100 µm). Statistical analyses were performed using one-way ANOVA for Figures A, C, D, and H, and two-way ANOVA for Figure F. Tukey’s post-hoc test was applied for multiple comparisons. Significant differences are indicated as follows: *P < 0.05, **P < 0.01, and ***P < 0.001.


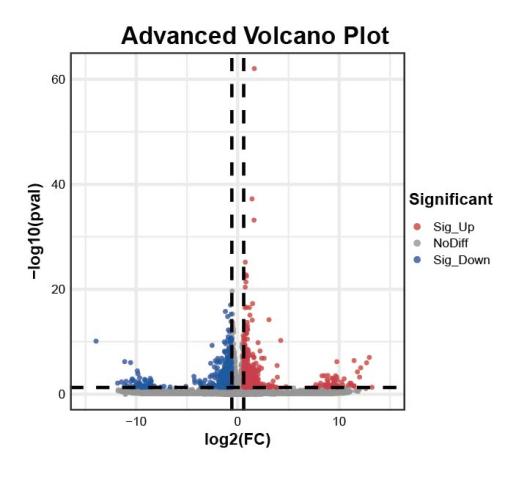


**Supplementary Figure 3.** Volcano plot of RNA-seq data from rat chondrocytes treated with either DAPA or DMSO.


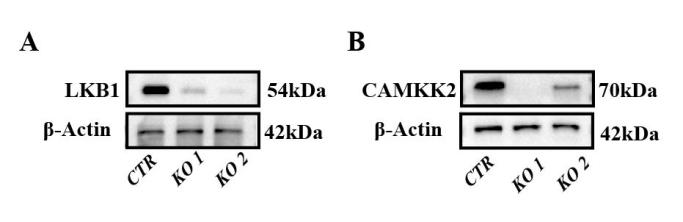


**Supplementary Figure 4. Detection of LKB1 and CAMKK2 Knockout Efficiency. (A)** Western blot verification of LKB1 knockout in C28 I2 cells. **(B)** Western blot verification of CAMKK2 knockout in C28 I2 cells.


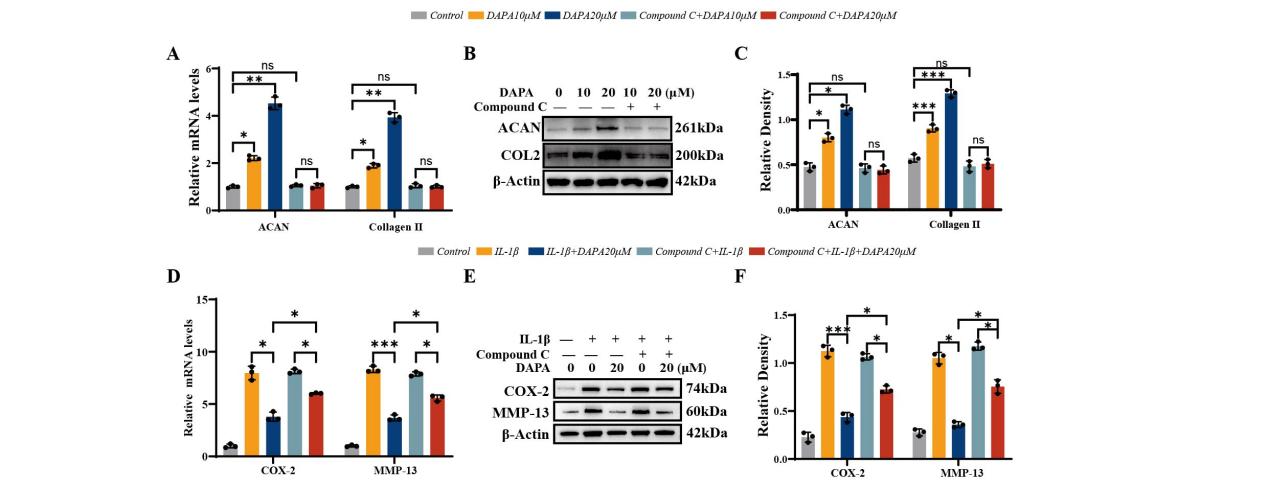


**Supplementary Figure 5. DAPA promotes chondrocyte anabolism via the AMPK signaling pathway. (A)** Human normal chondrocytes were pretreated with Compound C for 1 hour before being treated with various concentrations of DAPA for 24 hours. RT-qPCR was performed to measure the mRNA expression levels of ACAN and COL-2. **(B-C)** Human normal chondrocytes were pretreated with Compound C for 1 hour before being treated with various concentrations of DAPA for 48 hours. Western blotting was used to detect the protein expression levels of ACAN and COL-2. Protein band intensities were quantified using Image J (n = 3). **(D)** Human normal chondrocytes were pretreated with Compound C for 1 hour before being treated with IL-1β and DAPA for 24 hours. RT-qPCR was performed to measure the mRNA expression levels of COX-2 and MMP-13. **(E-F)** Human normal chondrocytes were pretreated with Compound C for 1 hour before being treated with IL-1β and DAPA for 48 hours. Western blotting was used to detect the protein expression levels of COX-2 and MMP-13. Protein band intensities were quantified using Image J (n = 3). Statistical analysis was performed using two-way ANOVA with Tukey's post hoc test. Significant differences are indicated as follows: *P<0.05 , **P<0.01, and ***P<0.001; ns: not significant.


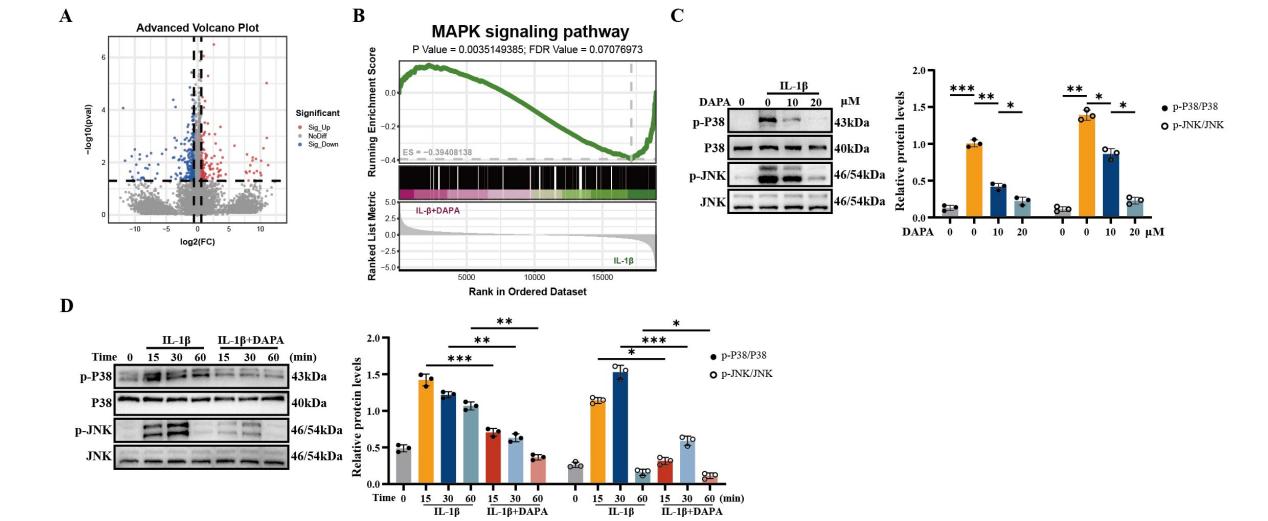


**Supplementary Figure 6. DAPA inhibits the IL-1β-mediated MAPK signaling pathway. (A)** Volcano plot of RNA-seq data from chondrocytes treated with DAPA and IL-1β. **(B)** Gene Set Enrichment Analysis (GSEA) plot of differentially expressed genes in the MAPK cascade pathway between IL-1β and DAPA+IL-1β treated chondrocytes. **(C)** In human chondrocytes treated with various concentrations of DAPA and IL-1β for 30 minutes, the phosphorylation levels of p38 and JNK proteins were assessed to evaluate pathway activity. Western blot results were quantified using ImageJ software (n=3). **(D)** In human chondrocytes treated with 10 μM DAPA and IL-1β at different time points, the phosphorylation levels of p38 and JNK proteins were measured to assess pathway activity. Western blot results were quantified using ImageJ software (n=3). Statistical analysis was performed using one-way ANOVA (Figure C) or two-way ANOVA (Figure D), followed by Tukey's post-hoc test for multiple comparisons. Significant differences are indicated as follows: *P < 0.05, **P < 0.01, and ***P < 0.001.


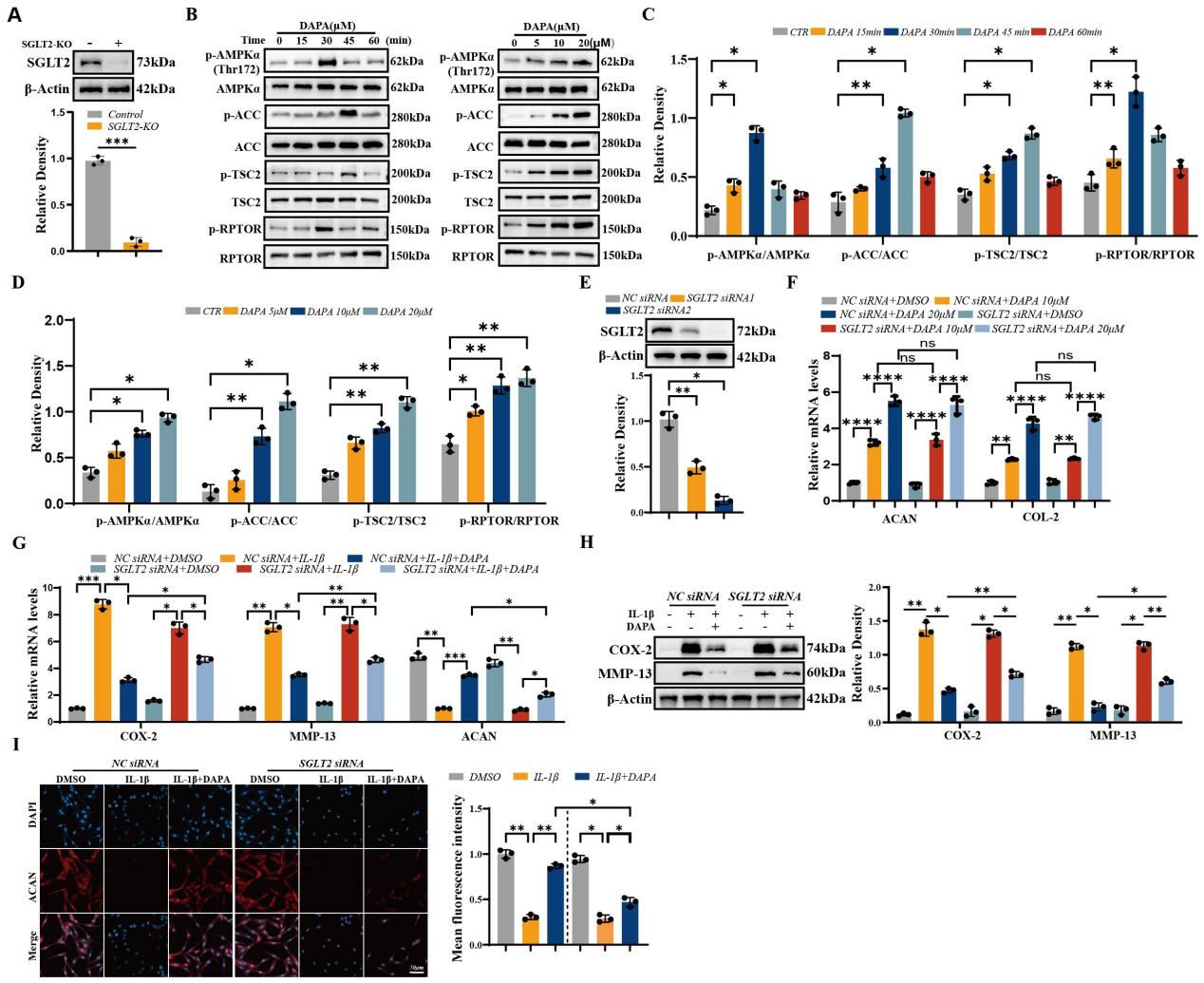


**Supplementary Figure 7. DAPA inhibits chondrocyte catabolism partially via SGLT2 targeting. (A)** Western blot confirmation of SGLT2 knockout in C28I2 cells. **(B)** Immunoblotting of AMPK, ACC, TSC2, and RPTOR signaling signalling activation in SGLT2 knockout C28I2 cells treated with DAPA (20 μM) for different lengths of time or different concentrations of DAPA (0, 5, 10, 20 μM) for 30 min. **(C-D)** Quantification of Western blotting results (n=3). **(E)** Immunoblotting analysis to examine the knockdown efficacy of siRNA against SGLT2 in human chondrocytes. Quantification of SGLT2 protein levels normalized to β-actin. **(F)** Human chondrocytes transfected with negative control siRNA (scRNAi) or SGLT2 RNAi were treated with or without DAPA (20 µM). RT-qPCR analysis of ACAN and COL-2 mRNA expression levels in the cells. **(G-I)** Human chondrocytes transfected with negative control siRNA (scRNAi) or SGLT2 RNAi were treated with or without IL1β (10 ng/mL) and with or without DAPA (20 µM). **(G)** RT-qPCR analysis of COX2, MMP13, and ACAN mRNA expression levels in the cells. **(H)** Representative immunoblots for COX2 and MMP13 proteins, with quantification normalized to β-actin protein levels. **(I)** Representative images of ACAN immunofluorescence staining and quantification of fluorescence intensity in chondrocytes. Statistical analysis was performed using independent t-tests or one-way/two-way ANOVA, as appropriate. Specifically, an independent t-test was used for the data in Figure A, one-way ANOVA for Figures C-E, and two-way ANOVA for Figures F-I. For ANOVA, Tukey's post-hoc test was applied to compare groups when a significant effect was found. Significant differences are indicated as follows: *P < 0.05, **P < 0.01, and ***P < 0.001.

**
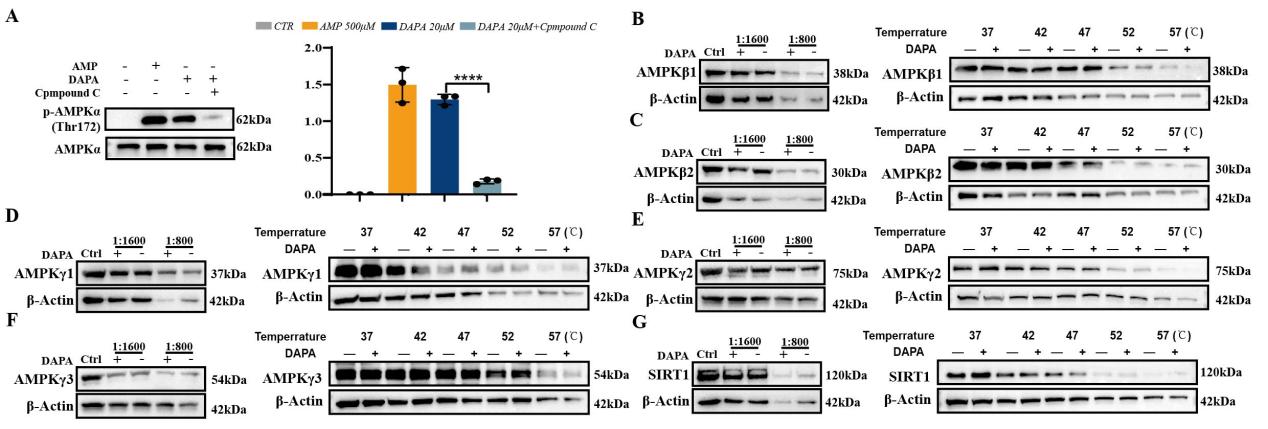
**

**Supplementary Figure 8. (A)** Analysis of in vitro AMPK kinase activity by immunoblotting. **(B-G)** Immunoblot analysis using anti-SIRT1, anti-AMPKβ and ,anti-AMPKγ antibody following DARTS and CETSA assay. Statistical analysis was performed using one-way ANOVA with Tukey's post hoc test. Significant differences are indicated as follows: ***P < 0.001.

**
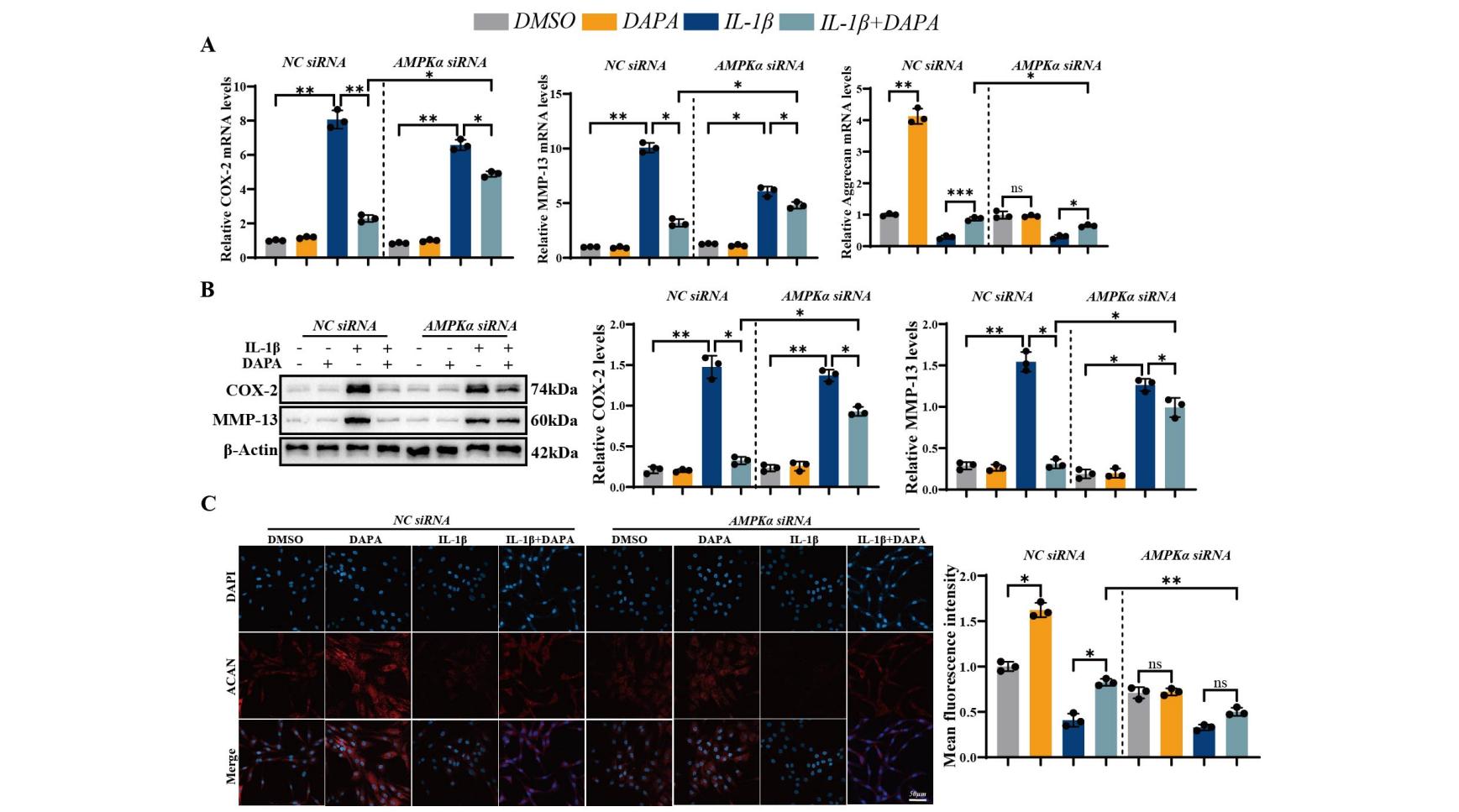
**

**Supplementary Figure 9. DAPA inhibits chondrocyte catabolism partially via AMPKα targeting.** Human normal chondrocytes transfected with negative control siRNA (scRNAi) or AMPKα RNAi were treated with or without IL1β (10 ng/mL) and with or without DAPA (20 µM). **(A)** RT-qPCR analysis of COX2, MMP13, and ACAN mRNA expression levels. **(B)** Representative immunoblots for COX2 and MMP13 proteins, with quantification normalized to β-actin protein levels. **(C)** Representative images of ACAN immunofluorescence staining and quantification of fluorescence intensity in chondrocytes. Statistical analysis was performed using two-way ANOVA with Tukey's post hoc test. Significant differences are indicated as follows: ns, not significant (p > 0.05), *P < 0.05, and **P < 0.01.


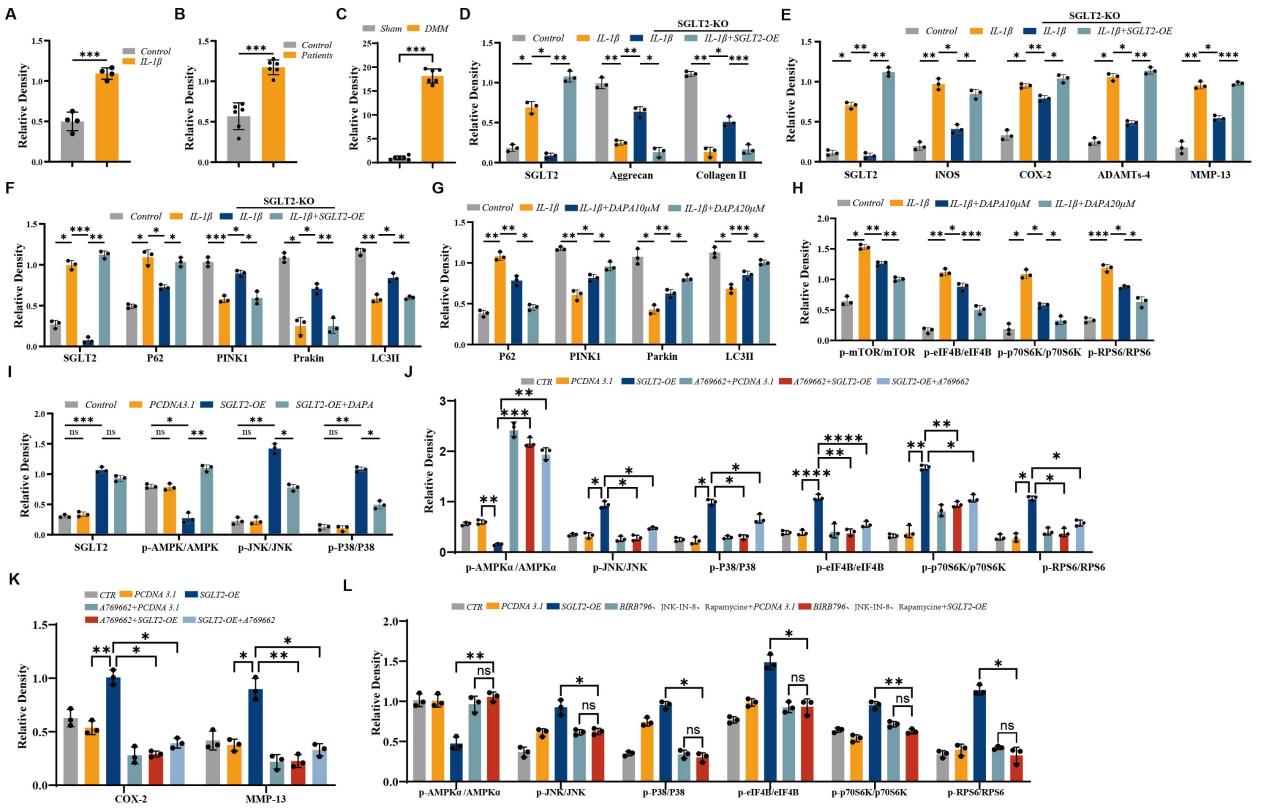


**Supplementary Figure 10. Quantitative analysis of Fig 7. (A)** Quantitative analysis of Western blot results shown in Fig 7A (n=4). **(B)** Quantitative analysis of Western blot results shown in Fig 7B (n=6). **(C)** Quantification of Immunohistochemical staining in Fig 7C. **(D)** Quantitative analysis of Western blot results shown in Fig 7G (n=3). **(E)** Quantitative analysis of Western blot results shown in Fig 7H (n=3). **(F)** Quantitative analysis of Western blot results shown in Fig 7K (n=3). **(G)** Quantitative analysis of Western blot results shown in Fig 7N (n=3). **(H)** Quantitative analysis of Western blot results shown in Fig 7O (n=3). **(I)** Quantitative analysis of Western blot results shown in Fig 7P (n=3). **(J-K)** Quantitative analysis of Western blot results shown in Fig 7Q (n=3). **(L)** Quantitative analysis of Western blot results shown in Fig 7R (n=3). Statistical comparisons were performed using unpaired t-tests (Fig. A–C), one-way ANOVA (Fig. D–I), or two-way ANOVA (Fig. J–L), as appropriate. For ANOVA-derived results, Tukey’s post-hoc test was applied to assess significant effects. Significant differences are indicated as follows: ns, not significant (p > 0.05), *P < 0.05, **P < 0.01, ***P < 0.001, and ****P < 0.0001.
