# Supplementary material for: Dapagliflozin regulates chondrocyte homeostasis and protects against osteoarthritis via targets AMPKα and SGLT2
Source: Cell Death Discov. 2026 Mar 19;12:217. doi: 10.1038/s41420-026-03016-y (PMC13172547; doi:10.1038/s41420-026-03016-y)
Supplement: Supplementary file 2 — Original Western Blots [file 41420_2026_3016_MOESM2_ESM.docx]

**Original Western Blots**

**lmages of the original western blots of Fig. 1**

**
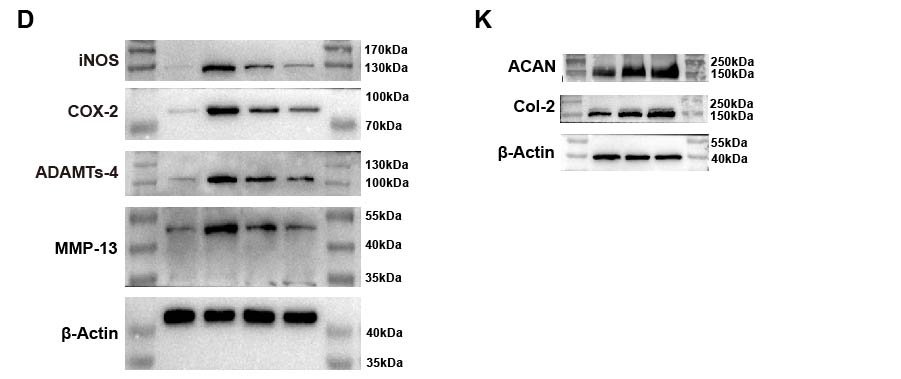
**

**lmages of the original western blots of Fig. 2**

**
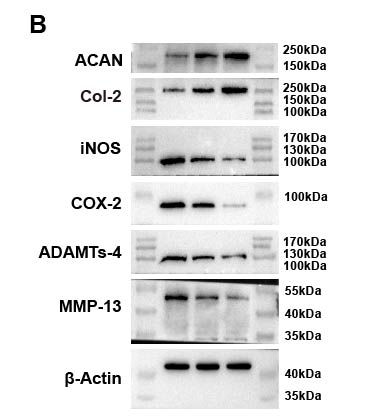
**

**lmages of the original western blots of Fig. 4**

**
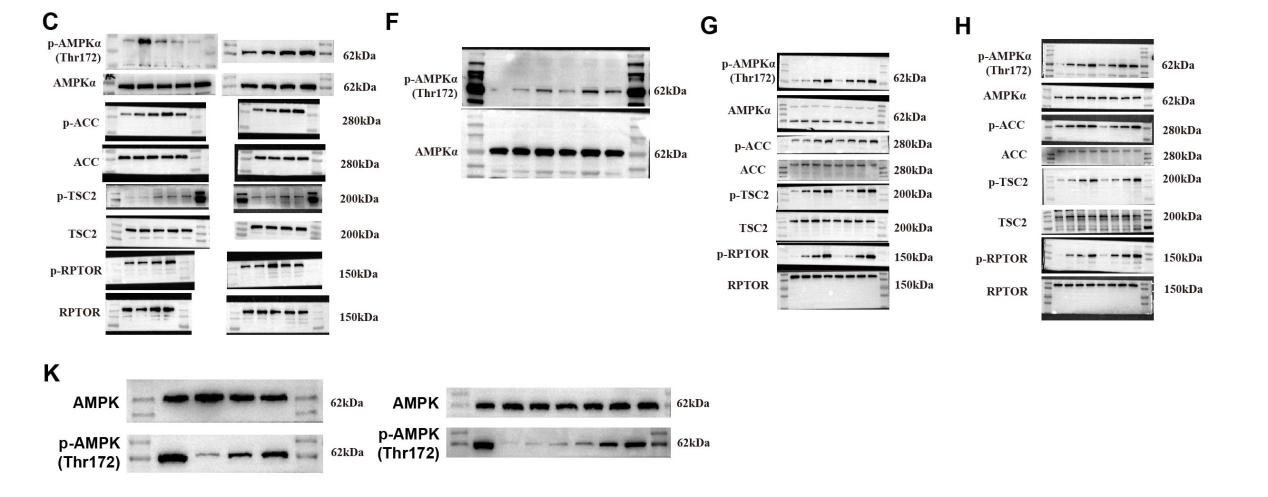
**

**lmages of the original western blots of Fig. 5**

**
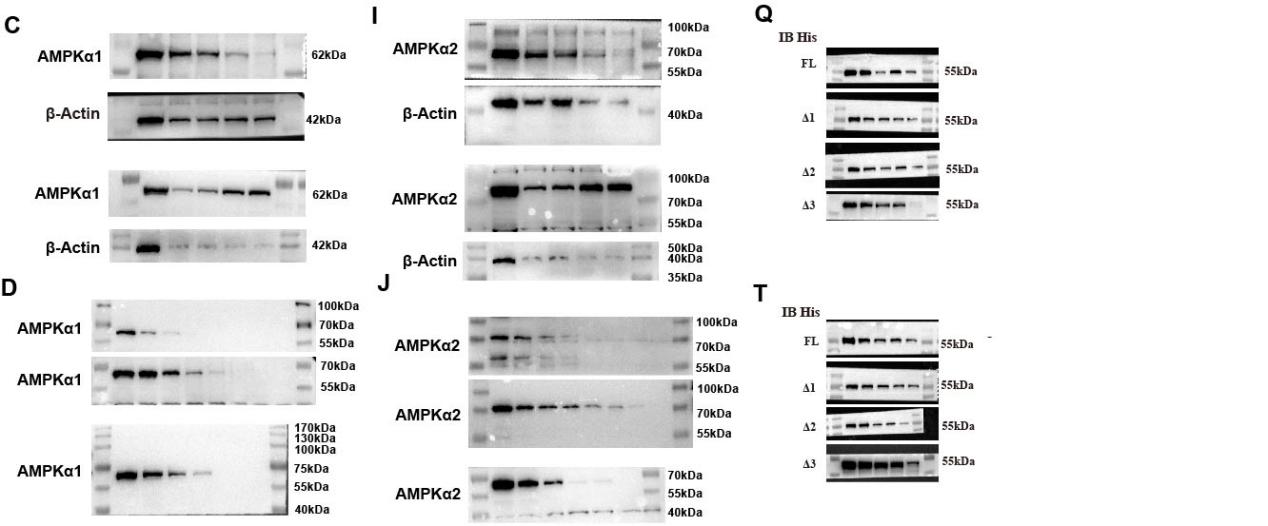
**

**lmages of the original western blots of Fig. 6**

**
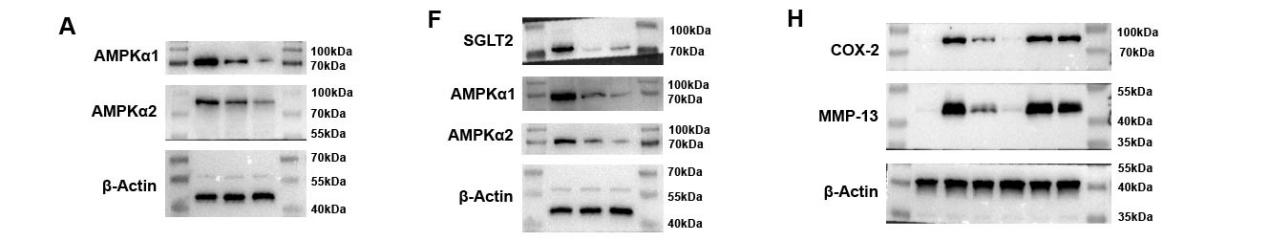
**

**lmages of the original western blots of Fig. 7**

**
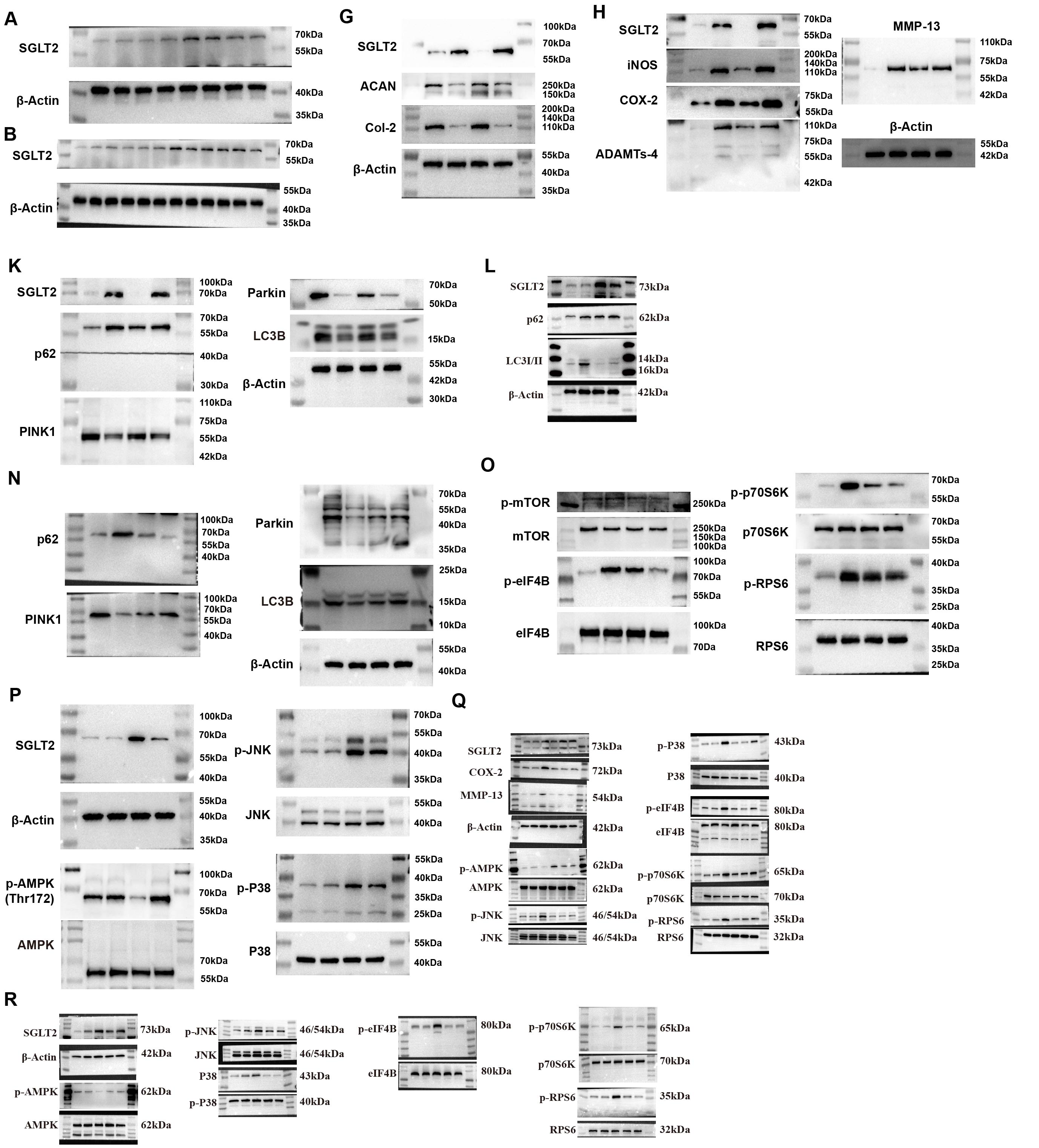
**

**lmages of the original western blots of Fig. S2**

**
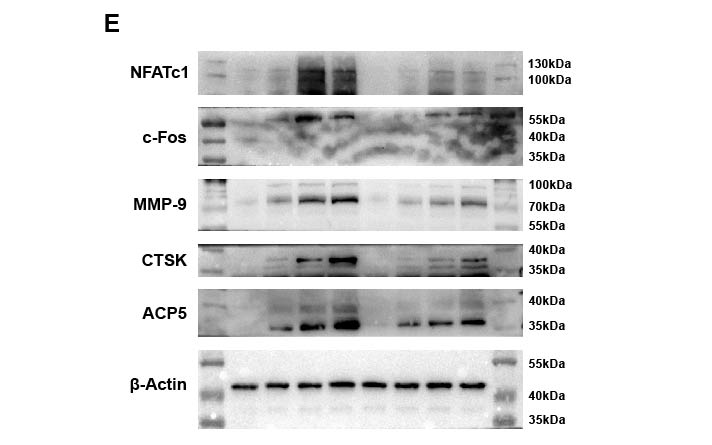
**

**lmages of the original western blots of Fig. S4**

**
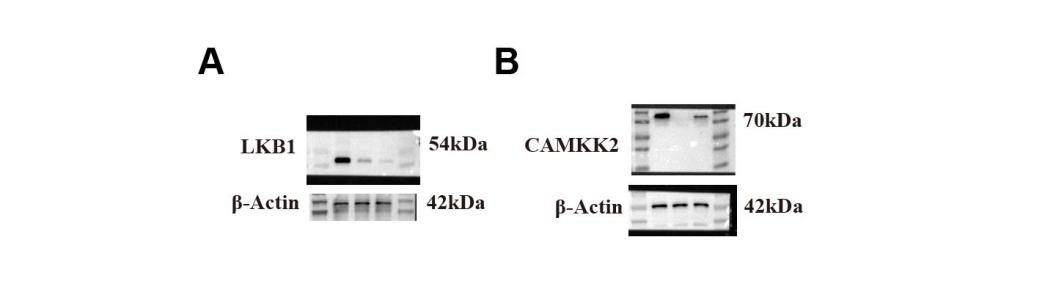
**

**lmages of the original western blots of Fig. S5**

**
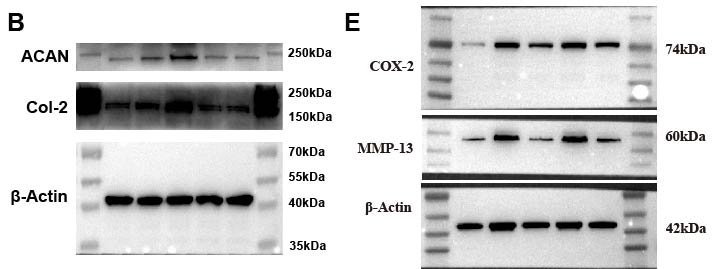
**

**lmages of the original western blots of Fig. S6**

**
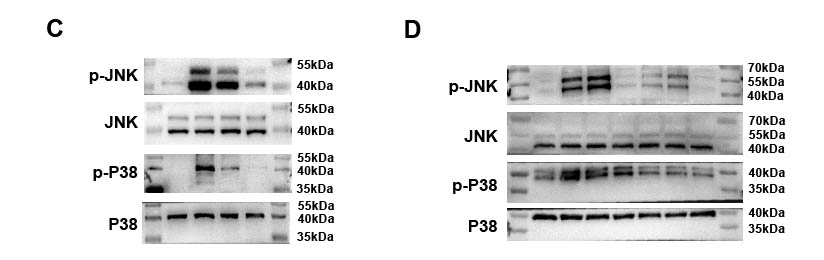
**

**lmages of the original western blots of Fig. S7**

**
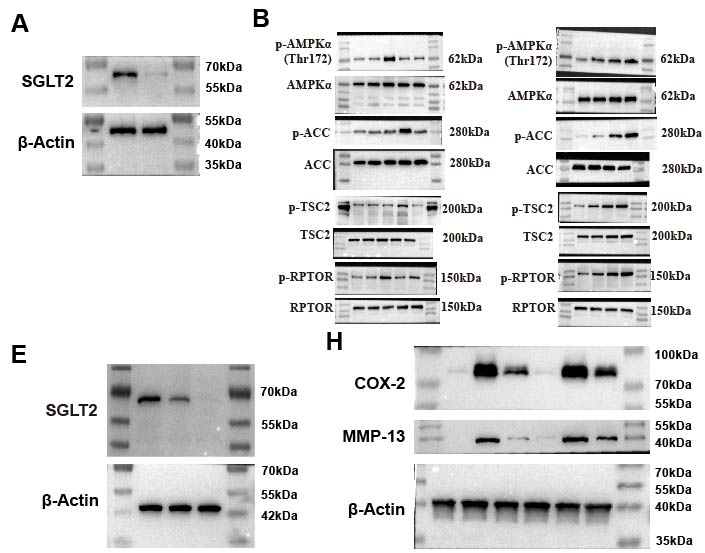
**

**lmages of the original western blots of Fig. S8**

**
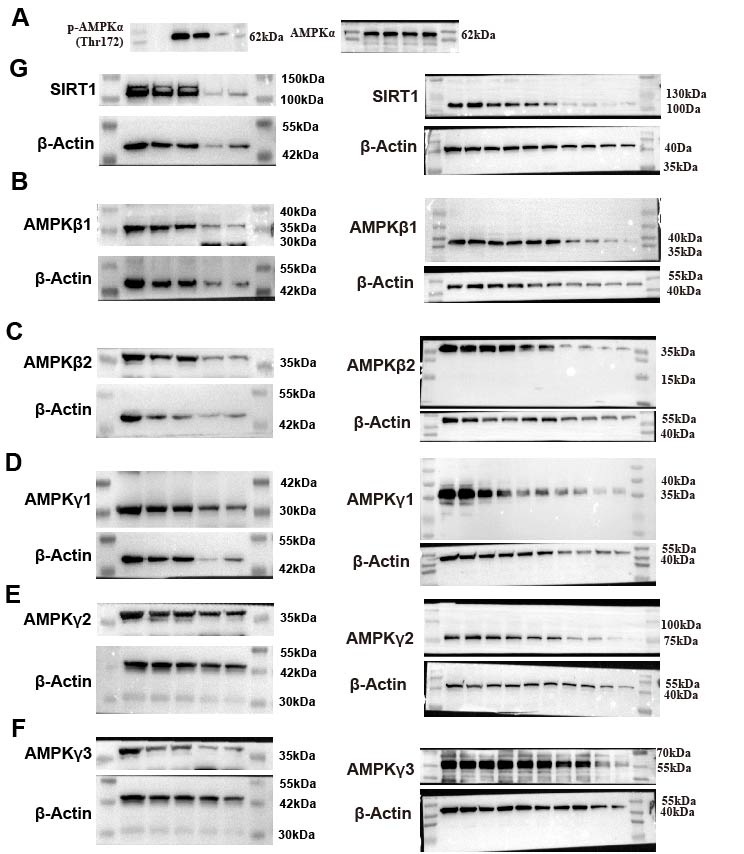
**

**lmages of the original western blots of Fig. S9**

**
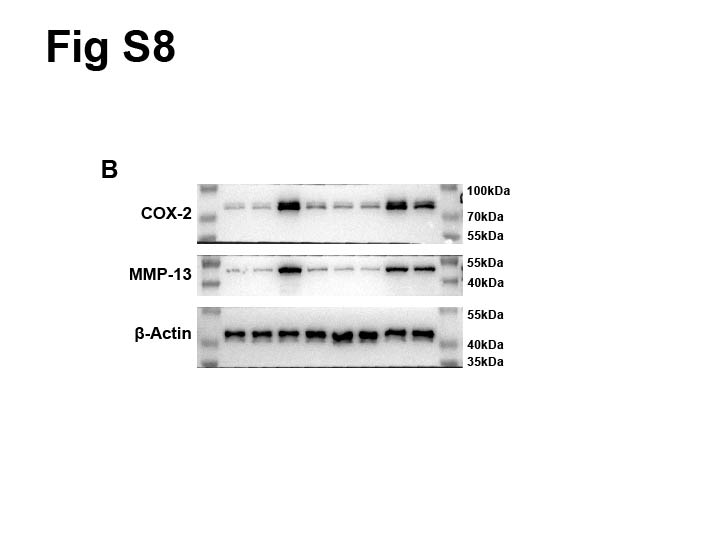
**
